# Nexus of Whey Proteins, Gut Dysbiosis, and Colonic Health

**DOI:** 10.1002/fsn3.71487

**Published:** 2026-02-05

**Authors:** Tolulope Joshua Ashaolu, Chi‐Ching Lee, Ozgur Tarhan, Ali Rashidinejad, Seid Mahdi Jafari

**Affiliations:** ^1^ Institute for Global Health Innovations Duy Tan University Da Nang Vietnam; ^2^ Faculty of Medicine Duy Tan University Da Nang Vietnam; ^3^ Department of Food Engineering, Faculty of Engineering and Natural Sciences Istanbul Sabahattin Zaim University Istanbul Turkey; ^4^ Department of Food Technology and Nutrition, Faculty of Technologies Klaipeda State University of Applied Sciences Klaipeda Lithuania; ^5^ Department of Food Engineering Uşak Üniversitesi Uşak Turkey; ^6^ Riddet Institute Centre of Research Excellence Massey University Palmerston North New Zealand; ^7^ Department of Food Materials and Process Design Engineering Gorgan University of Agricultural Sciences and Natural Resources Gorgan Iran; ^8^ Halal Research Center of IRI, Iran Food and Drug Administration Ministry of Health and Medical Education Tehran Iran

**Keywords:** bioactive peptides, colonic health, gut dysbiosis, gut microbiota, metabolic disorders

## Abstract

The gut microbiota is essential for colonic health, and its imbalance (dysbiosis) is linked to conditions like inflammatory bowel disease and metabolic disorders. Whey proteins (WPs), including β‐lactoglobulin, α‐lactalbumin, glycomacropeptide, and lactoferrin, possess antimicrobial, immunomodulatory, and prebiotic‐like properties that may help restore microbial balance. Beyond modulating the microbiome, WPs play a significant role in reinforcing intestinal barrier integrity and regulating host metabolism. This review summarizes evidence from in vitro, in vivo, and clinical studies showing WPs can enhance beneficial bacteria (e.g., *Bifidobacterium*, *Lactobacillus*) while suppressing harmful ones. Furthermore, WP supplementation has been shown to alleviate dysbiosis‐related conditions such as colitis, obesity, and allergies by improving microbial diversity, enhancing short‐chain fatty acid production, strengthening the mucosal barrier, and modulating immune responses. However, the effects vary depending on WP composition, processing, and individual microbiota. Despite encouraging results, knowledge gaps remain regarding optimal dosing and long‐term impacts. Overall, WPs show promise as functional food components and potential therapeutic agents for promoting colonic health, metabolic homeostasis, and gut barrier function, but more research is needed to refine their clinical application.

## Graphical abstract

Whey proteins possess antimicrobial and prebiotic properties that modulate gut microbiota by promoting beneficial bacteria like *Bifidobacterium* while suppressing pathogens. This modulation enhances short‐chain fatty acid production and strengthens intestinal barrier integrity, potentially alleviating dysbiosis‐related conditions such as colitis and metabolic disorders. Despite these benefits, clinical outcomes vary based on protein composition and individual microbiota, indicating a need for further research to optimize therapeutics.

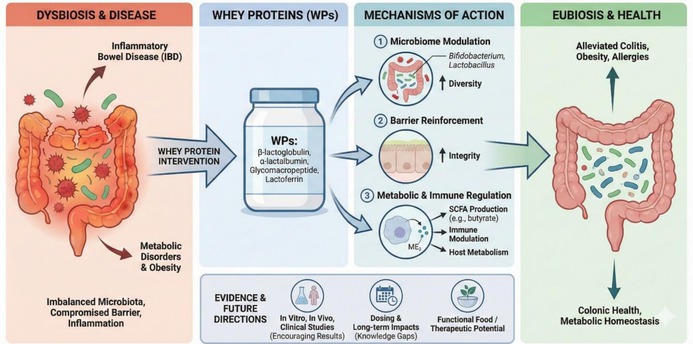

**Abbreviations**
AA: amino acid
ALA: α‐lactalbumin
BAP: bioactive peptide
BCAA: branched‐chain amino acid
BCFA: branched‐chain fatty acid
BLF: bovine lactoferrin
BLG: β‐lactoglobulin
BSA: bovine serum albumin
DSSS: dextran sulfate sodium salt
FMT: fecal microbiota transplantation
GI: gastrointestinal
GLMP: glycomacropeptide
GM: gut microbiota
GMd: gut microbiota dysbiosis
GOS: galactooligosaccharides
IBD: inflammatory bowel disease
Ig: immunoglobulin
IBS: irritable bowel syndrome
IFN‐γ: interferon gamma
IL: interleukin
LAB: lactic acid bacteria
LF: lactoferrin
LPS: lipopolysaccharide
LP: lactoperoxidase
LZ: lysozyme
NP: nanoparticle
PKU: phenylketonuria
POS: polycystic ovary syndrome
rh‐LF: recombinant human lactoferrin
SCFA: short‐chain fatty acid
sIgA: secretory immunoglobulin A
TLR: toll‐like receptor
TNF‐α: tumor necrosis factor alpha
WP: whey protein
WPC: whey protein concentrate
WPH: whey protein hydrolysate
WPI: whey protein isolate

## 1 Introduction

The human gastrointestinal (GI) tract harbors a complex microbial ecosystem of over 10^14^ cells across thousands of species whose collective gene repertoire far exceeds that of the host (Afzaal et al. 2022). These gut microbes engage in mutualistic activities that drive digestion and host metabolism, reinforce epithelial barrier integrity, and regulate immune and inflammatory pathways (Afzaal et al. 2022; Hou et al. 2022). The gut microbiota (GM) is often termed a “vital organ” due to its extensive crosstalk with host systems via neural, endocrine, immune, and metabolic axes. It is a complex ecosystem of bacteria, fungi, viruses, and archaea, and plays a pivotal role in maintaining colonic and systemic homeostasis. GM facilitates nutrient metabolism, synthesizes essential vitamins (e.g., B_12_ and K), and modulates immune responses through interactions with host epithelial cells and lymphoid tissues (Madsen et al. 2017; Hrncir 2022). A balanced GM also produces short‐chain fatty acids (SCFAs) like butyrate, which nourish colonocytes, reduce inflammation, and strengthen the intestinal barrier (Monteiro et al. 2016). Dysregulation of these functions is increasingly linked to pathologies e.g., inflammatory bowel disease (IBD), colorectal cancer, and metabolic disorders, underscoring the GM's critical role in health (Hrncir 2022; Govindarajan 2024). Diet and other environmental factors strongly shape GM composition, so perturbations of this community can disrupt digestive function and systemic homeostasis (Hou et al. 2022).

GM dysbiosis (GMd) is broadly defined as an imbalance in the gut microbial community, typically marked by the loss of beneficial taxa, e.g., *Faecalibacterium prausnitzii* , and the overgrowth of opportunistic pathogens/pathobionts (e.g., *Streptococcaceae*, *Proteobacteria*) (Hrncir 2022; Govindarajan 2024; Safarchi et al. 2025). Such compositional shifts reduce microbial diversity and undermine normal GM functions. It can compromise the mucus layer, increase intestinal permeability, and trigger inflammation through lipopolysaccharide (LPS) translocation and pro‐inflammatory cytokine release (e.g., TNF‐α), fueling chronic inflammation (Ahmad Kendong et al. 2021). Importantly, these microbial imbalances have been strongly linked to colonic pathology. For example, specific dysbiotic signatures are associated with IBD and colorectal cancer (Ahmad Kendong et al. 2021; Safarchi et al. 2025). Also, dysbiosis‐driven mucin degradation by *Akkermansia muciniphila* has been linked to Crohn's disease, while *Ruminococcus* species are associated with polyp formation (Quesada‐Vázquez et al. 2020; Welham et al. 2023). Thus, maintaining a balanced (eubiotic) GM is considered critical for colonic health and preventing disease.

Whey proteins (WPs) are the soluble protein fractions of milk, predominantly composed of β‐lactoglobulin (BLG) and α‐lactalbumin (ALA), along with minor components, e.g., bovine serum albumin (BSA), immunoglobulins (Igs), and lactoferrin (LF) (Quintieri et al. 2025). They are a rich source of bioactive peptides (BAPs), essential amino acids (AAs), and branched‐chain AAs (BCAAs), giving whey a high biological value. Structurally, WP comprises BLG (~65%), ALA (~25%), glycomacropeptide (GLMP), LF, and Igs, each contributing unique biochemical properties (Boscaini et al. 2023; Rackerby et al. 2024). Beyond basic nutrition, digestion or fermentation of WP releases BAPs with diverse physiological activities. Numerous WP‐derived peptides exhibit antimicrobial, antioxidant, and immunomodulatory properties and can act directly within the gut lumen (Quintieri et al. 2025). For example, GLMP—a casein‐derived peptide abundant in whey—has been shown to selectively promote the growth of beneficial gut bacteria (*Bifidobacterium, Lactobacillus*) while inhibiting pathogens (Rackerby et al. 2024). These prebiotic‐like effects suggest that WP or its components may help restore microbial balance and mitigate dysbiosis (Zhao and Ashaolu 2020; Zhao et al. 2022; Zhao et al. 2023). In addition, recent studies also highlight its ability to reshape microbial communities in high‐fat diets by increasing *Bacteroidetes* and reducing *Firmicutes*, a shift associated with improved metabolic health (Monteiro et al. 2016; Zhu et al. 2025).

In this review, we aim to clarify the interplay between dietary WPs, gut microbial balance, and colonic health. Specifically, we explain the biochemical composition and bioactive properties of WPs relevant to the gut, delineate the consequences of WPs on GMd and colonic physiology, and synthesize current evidence on how whey‐derived factors influence GM composition and colonic outcomes. By integrating these topics, the review will highlight how WP‐based interventions might support colonic homeostasis and will identify knowledge gaps in understanding this nutritional–microbial nexus.

## 2 Whey Protein Composition

WP is a mixture of proteins derived from whey, the liquid byproduct of milk when cheese is made. Whey consists of around 20% milk protein, with BLG, ALA, BSA, and Igs being the major proteins. WP is a heterogeneous mixture of components whose exact composition differs depending on the processing process. The WP fraction is intricate, consisting of numerous proteins imparting distinct nutritional and functional characteristics. BLG is the most concentrated protein in whey and makes up about 50%–55% of WPs. It is a globular protein that contains essential AAs and accounts for some milk allergies in sensitive people. It has an important function of binding fat‐soluble vitamins and minerals and can increase their bioavailability upon digestion (de Paula et al. 2024). ALA accounts for approximately 20%–25% of WP and is especially high in tryptophan, an essential AA that serves as a precursor for the neurotransmitter serotonin. ALA is also involved in the synthesis of lactose in the mammary gland and has been researched for its theoretical involvement in sleep quality and mood control due to its tryptophan content. BSA represents 5%–10% of WPs and has the ability to bind fatty acids and other minor molecules. Igs, including IgG, IgA, and IgM, constitute 10%–15% of WPs and are credited with immune‐supportive activity, particularly valuable in colostrum, the first milk post‐parturition. WP also has some minor but biologically important components, such as LF presenting in 1%–2% of WPs, having antimicrobial and iron‐binding properties, and lactoperoxidase (LP) in 0.5% of WPs, an enzyme with antimicrobial action. GLMP accounts for 10%–15% of WPs and is being investigated for potential prebiotic activity (Mehra et al. 2021).

The AAs profile of WPs contains very high concentrations of BCAAs. Leucine, which makes up 10%–14% of total AAs, is particularly notable in stimulating muscle protein synthesis and isoleucine and valine round out the BCAA family. WP is also rich in glutamine, critical to intestinal health; cysteine, helpful in stimulating antioxidant production; and lysine, which plays a role in the growth and repair of tissue. The protein composition of human milk WPs differs markedly from bovine WPs. While BLG constitutes the predominant protein (50%–55%) in bovine whey, it is completely absent in human milk. This absence is significant because BLG is a major allergen in cow's milk that can trigger adverse reactions in sensitive individuals (Dullius et al. 2018). The immunological components in human milk whey are proportionally more abundant and diverse than in bovine milk. Human milk contains substantially higher concentrations of secretory Ig A (sIgA), which provides crucial passive immunity to infants, while bovine milk contains predominantly IgG. The concentration of lysozyme (LZ) in human milk is approximately 3000 times higher than in bovine milk, highlighting its importance in gut protection in infant humans. LZ is a cell wall cleaver in bacteria and functions in synergism with other BAPs to shape the developing microbiome and protect against potential pathogens. Such an extreme difference indicates species‐specific antimicrobial strategies developed over evolution (Saadi et al. 2024).

Commercially, WP is manufactured in three primary forms. WP concentrate (WPC) contains 30%–80% of protein content with a little lactose, fat, and minerals. WP isolate (WPI) is a more processed form with approximately 90% protein content and minimal lactose, fat, and minerals. Besides, WP hydrolysate (WPH) is produced from predigested and partially hydrolyzed whey, allowing for faster absorption with a bitter taste. Both nutritional and functional attributes of WPs render it useful as a protein supplement and an ingredient with the ability to modulate metabolic processes, including GM. BAPs produced by the processing or digestion of WPs exhibit a range of physiological activities whose exploration is still developing through research (Vasconcelos et al. 2021).

## 3 Impact of Whey Proteins on Microbial Metabolism

**FIGURE 1:**
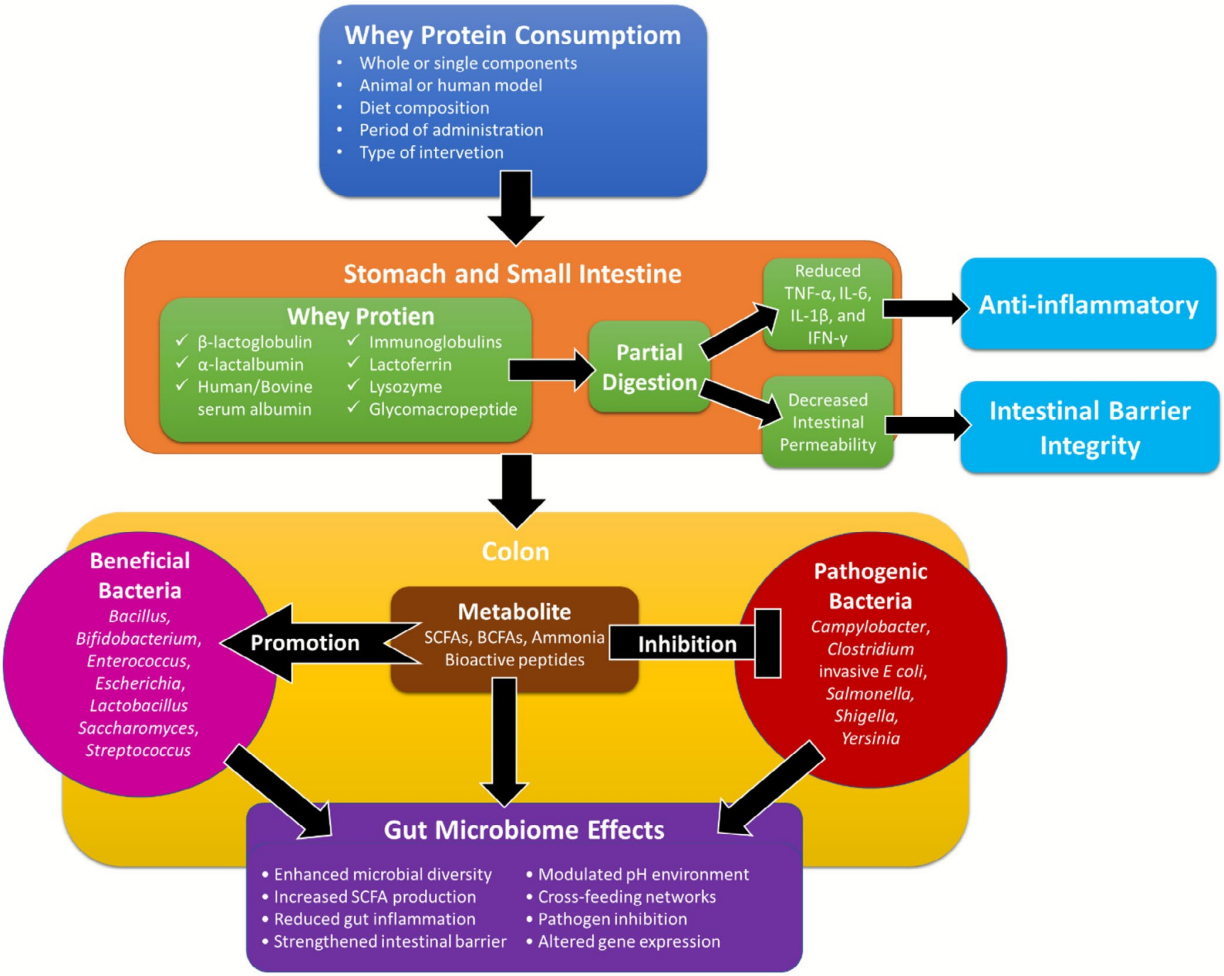
Influence of whey proteins on gut microbial metabolism and community structure. BCFAs, branched‐chain fatty acids; IFN‐γ, interferon gamma; IL‐1β, interleukin 1β; IL‐6, interleukin 6; SCFA, short‐chain fatty acids; TNF‐α, necrosis factor alpha.

WPs are a heterogeneous group of bioactive molecules that may exert a profound effect on the microbial community structure and metabolism in the gut through a number of mechanisms (Figure 1). The proteins, reaching the colon in a partially digested state, act as substrates for proteolytic bacteria. The fermentation yields metabolites such as branched‐chain fatty acids (BCFAs), ammonia, phenols, and hydrogen sulfide. Unlike carbohydrate fermentation, which produces beneficial SCFAs, protein fermentation metabolites can potentially create a more alkaline environment that favors pathogenic bacteria over beneficial commensals (Sánchez‐Moya et al. 2017). WPs also contain BAPs generated on digestion and have intense effects on microbial populations. LF has antimicrobial properties against pathogenic bacteria with the ability to promote the growth of beneficial organisms such as *Bifidobacteria*. GLMP has prebiotic properties, selectively promoting the growth of *Bifidobacteria* and *Lactobacilli*. Peptides from BLG can also affect bacterial adhesion to intestinal mucosae, thereby affecting the microbial profile (Lee et al. 2024).

Experiments demonstrate that supplementation with WP exerts a broad range of significant effects on microbial community composition. Classical responses enrich health‐beneficial microbes, e.g., *Bifidobacterium* and *Lactobacillus*, and SCFA‐producers such as *Roseburia* and *Faecalibacterium*. At the same time, decreases in potentially less desirable bacteria, such as certain *Bacteroides* and *Clostridium* species, as well as lower counts of proteolytic bacteria linked to inflammatory metabolites, have been reported (Boscaini et al. 2023; Reis 2021). WP metabolism creates intricate cross‐feeding networks whereby metabolites produced by one bacterial species are used as substrates by others. Some peptides released by the degradation of WPs by primary degraders, for example, may become substrates for growth by secondary fermenters, creating metabolic interdependencies that determine community structure (Lee 2025a). Such networked metabolic processes contribute greatly to the resilience and stability of the gut ecosystem. Daily supplementation with WPs causes adaptive responses within microbial metabolism that can be accountable for long‐term alteration of gut function. Adaptations encompass the improvement of proteolytic enzyme secretion by some bacteria, gene expression alteration of AA transporters and metabolizers, and altered quorum sensing processes influencing biofilm formation and inter‐bacterial communication. The alterations show the plasticity and responsiveness of GM to dietary intervention with WPs (Cava et al. 2024; Sepandi et al. 2022). The influence of different components of WPs on GM, in vivo studies and clinical trials as reported in some studies, are presented in Table 1.

**TABLE 1:** Influence of different components of whey proteins (WPs) on gut microbiota (GM); in vivo studies and clinical trials.

Major gut microbiota	Human participants or animal models	Purpose of study	Experimental design	Significant Outcomes	References
ß‐lactoglobulin (BLG)					
*Clostridium butyricum* CGMCC0313‐1	Male BALB/c mice aged 6–8 weeks	A mouse model of food allergy was used to investigate if *Clostridium butyricum* CGMCC0313‐1 may alleviate intestine anaphylaxis caused by BLG	Mice were sensitized orally with 20 mg BLG plus 10 μg cholera toxin or only CTX on days 7, 14, and 21, and followed by oral administration of 100 mg BLG challenge on day 28. Mice also received *C. butyricum* feeding orally from day 1 to day 21 or from day 22 to day 28	*Clostridium butyricum* significantly decreased the symptoms of intestinal anaphylaxis by enhancing Secretory IgA and CD4+ CD25+ Foxp3Treg cells. The imbalance in the expression of Th1/Th2 and Th17/Treg transcription factors was also rectified	Zhang et al. (2017)
*Lactobacillus acidophilus* KLDS 1.0738	Female BALB/c mice aged 5–6 weeks with an average weight of 23 ± 0.92 g	The relationship between Th17‐dominated BLG allergy prevention and *L. acidophilus* capacity to regulate miRNA expression was Investigated in an allergic mouse model	Mice in BLG allergic group and *L. acidophilus* ‐treated group mice were both intraperitoneally injected with 0.2 mg BLG on days 7, 14, and 21. Mice in *L. acidophilus* ‐treated groups were intragastrically treated with bacterial suspension three times a week from days 1 to 28. All mice were orally challenged with BLG on day 28, except the normal group	Live *L. acidophilus* treatment significantly inhibited Th17 proliferation and hypersensitivity reactions. Furthermore, live *L. acidophilus* decreased the expression of four miRNAs. The reduction in IL‐17 and RORγt mRNA expression was directly linked to the reduced expression of miRNAs in the group treated with *L. acidophilus*	Wang et al. (2018)
*Alistipes* sp. *Bacteroides* sp. *Blautia* sp. *Bifidobacterium* sp. *Faecalibacterium* sp. *Lachnoclostridium* sp. *Lachnospira* sp. *Lachnospiraceae* sp. *Oscillospiraceae* sp. *Roseburia* sp. *Sutterella* sp.	Two strains of female BALB/c and C57Bl/6 mice aged 3–6 weeks	The identification of new biomarkers and the development of creative preventative and therapeutic approaches depend on an understanding of the fundamental mechanisms underlying IgE‐mediated cow's milk allergy	4‐week‐old mice received oral gavages containing BLG and cholera toxin once a week for 6 weeks. Intragastrical administration of BLG was in PBS or oil emulsion, followed by intraperitoneal injection of sodium salicylate. The latter protocol was performed 5 times a week for 3 weeks	Several metabolic changes were caused by microbes during mouse sensitization, particularly in the metabolites of bile acid, energy, and tryptophan, which came before allergic inflammation	De Paepe et al. (2024)
*Alistipes* sp. *Bacteroides* sp. *Blautia* sp. *Bifidobacterium* sp. *Faecalibacterium* sp. *Lachnoclostridium* sp. *Lachnospira* sp. *Lachnospiraceae* sp. *Oscillospiraceae* sp. *Roseburia* sp. *Sutterella* sp.	22 and 21 children with and without IgE‐mediated cow's milk allergy,15 children with IgE‐other, and 23 healthy children	The metabolic changes that underlie IgE‐mediated cow's milk allergy in children were determined	Polar metabolomics, lipidomics, and 16S rRNA meta‐sequencing were conducted after fecal and urine sample collection	In children following elimination diets, GM dysbiosis (GMd) stimulated a persistent low‐grade inflammation that preceded allergic inflammation	De Paepe et al. (2024)
α‐lactalbumin (ALA)					
*Lactiplantibacillus plantarum* HM‐22	ALA‐induced allergic mice	The impact of *L. plantarum* HM‐22 on intestinal inflammation and intestinal microbiota was examined in mice with ALA‐induced allergies	ALA‐induced allergic mice were administered *L. plantarum* HM‐22 intragastrically for 5 weeks at both low and high dosages	*L. plantarum* HM‐22 raised levels of interleukin‐10, interferon‐γ, and transforming growth factor‐β while lowering levels of total IgE and the pro‐inflammatory factor interleukin‐4. The intestinal microbes of ALA‐induced allergic mice also improved, the crypt structure of their colon tissues changed, goblet cells decreased, and the phenomenon of numerous inflammatory corpuscles that appeared was improved and alleviated. The probiotic *L. plantarum* HM‐22 can prevent allergies by altering the gut flora	Jiang et al. (2021)
*Alloprevotella* *Bacteroides* *Blautia* *Eubacterium* *Lachnoclostridium* *Lachnospiraceae* *Lactobacillu* *Marvinbryantia* *Roseburia* *Ruminococcaceae* *Ruminiclostridium* *Ruminococcus* *Peptoclostridium* *Prevotellaceae* *Turicibacter*	Male SD rats aged 4 weeks with an average weight of 150 g	The effects of various ALA concentrations on the variety and structure of gut flora and the optimal concentration of ALA to add to infant formula were determined under the male rat model	SD rats were divided into four groups and received 200, 100, and 20 mg/kg of ALA and an equivalent amount of normal saline as a control group orally. After 14 and 28 days, colonic feces were aseptically removed	The species richness, diversity, and structure of GM were enhanced by 200 and 100 mg/kg of ALA, with an impact comparable to that of breast milk. The relative abundance of *Lactobacillus*, *Blautia*, and *Akkermansia* was decreased by the high level of ALA. It was suggested that the medium dose of 100 mg/kg of ALA was optimal for controlling intestinal microbiota since it improved gut microbial diversity, maintained or raised the relative abundance of gut probiotics, and decreased the levels of gut pathogenic bacteria	Menghan et al. (2020)
*Lachnospiraceae* *Clostridiaceae* *Clostridiales, Clostridium* spp. *Clostridiaceae* *Enterobacteriaceae* *Streptococcus*, *Streptomyces*	40 Danish Landrace × Large White × Duroc newborn piglets had been born by Cesarean delivery from two sows at 90% gestation with 106 days. All piglets were placed in oxygenated, heated incubators at 37°C after being resuscitated	Supplementing with WP concentrate (WPC) enriched with ALA was used to investigate the development of newborn pigs	Dilute bovine milk, or this milk supplemented with WPC with normal or high levels of ALA, was administered to preterm piglets delivered by cesarean section. On Day 19, tissues were collected to evaluate clinical, intestinal, immunological, and cognitive criteria	High levels of ALA tended to exhibit a higher growth rate, a quicker meal transit time, increased bone mineral content, more colon microbial diversity, and a higher abundance of certain bacteria and microbial metabolites. WPC supplementation in milk could enhance relative organ weights, blood amino acids (AAs), blood neutrophil function, and microbial metabolites	Nielsen et al. (2020)
*Alistipes* *Bacteroides* *Helicobacter* *Muribaculaceae* *Prevotellaceae* *Parabacteroides* *Rikenellaceae* *Ruminococcaceae*	Spontaneously hypertensive rats	The peptide VGINYW and ALA hydrolysates under 3 kDa were evaluated for their antihypertensive impact, and the potential mechanism was demonstrated in spontaneously hypertensive rats	Spontaneously hypertensive rats were administered with 5 mg/kg VGINYW and 100 mg/kg ALA hydrolysates	In addition to significantly lowering the systolic blood pressure of the SHRs, VGINYW and ALA hydrolysates may also significantly reduce oxidative stress within the spontaneously hypertensive rats. More importantly, by restoring the diversity of GM and modulating the critical floras that produce short‐chain fatty acids (SCFAs), the gavage of VGINYW and ALA hydrolysates could potentially treat intestinal microbiota dysbiosis linked to hypertension	Xie et al. (2022)
*Alistipes* *Bacteroides* *Helicobacter* *Muribaculaceae* *Prevotellaceae* *Parabacteroides* *Rikenellaceae RC9* *Ruminococcaceae UCG‐014*	Forty Male ICR mice with an average weight of 26 ± 3 g	The isolated gastrointestinal (GI) hydrolysates of ALA were used to examine the possible anti‐hyperuricemic and nephroprotective effects on hyperuricemic mice	The mice were administered 300 mg/kg potassium oxonate and 300 mg/kg hypoxanthine orally every day to induce hyperuricemia	GM assessments showed that ALA hydrolysate treatment suppressed the proliferation of genera linked to inflammation and hyperuricemia while increasing the abundance of certain producers of mice fecal SCFAs. By reducing oxidative stress and inflammation and modifying GM in hyperuricemic rats, the isolated GI hydrolysates of α‐lactalbumin exhibited anti‐hyperuricemia and nephroprotective effects	Xie et al. (2023)
*Alistipes* *Bacteroides* *Blautia*, *Lachnospiraceae NK4A136 group* *Muribaculaceae* *Rikenellaceae RC9 gut group*	High‐fat diet‐induced mice	The potential mechanisms by which ALA peptide Asp‐Gln‐Trp improved GMd and insulin resistance were examined in mice with nonalcoholic fatty liver disease brought on by a high‐fat diet	Mice were induced nonalcoholic fatty liver disease under a high‐fat diet	ALA peptide Asp‐Gln‐Trp treatment increased the ratio of *Bacteroides* to *Firmicutes* in the GI tract, decreased the relative abundance of pathogenic bacteria, and increased the relative abundance of bacteria that produce SCFAs. Treatment with ALA peptide increased the synthesis of SCFAs, which in turn reduced inflammation and enhanced the intestinal barrier. This treatment may modify GM composition and stimulate the IRS1/PI3K/Akt and PPARα pathways	Chen et al. (2022)
*Bifidobacterium* *Lactobacillus*	10 female patients with clinical signs of polycystic ovary syndrome (POS)	The capacity of ALA was accessed on the effects in the treatment of POS to restore GMd	10 women under a 30‐day oral administration of ALA with 300 mg/twice a day	ALA may have a role in increasing the growth of bacteria that promote health while restricting the growth of possible pathogens. The findings may help restrict or repair intestinal dysbiosis associated with POS by establishing ALA as a legitimate substance with prebiotic effects	Alessandri et al. (2024)
*Lactobacillus acidophilus* *Bifidobacterium short* *Bifidobacterium longum* *Bifidobacterium infantis*	37 obese and dysmetabolic patients were men and women between the ages of 25 and 65 with a body mass index (BMI) between 30 and 40 and fasting blood glucose levels between 100 and 125 mg/dL	The effects of a hypocaloric Mediterranean diet combined with supplements of myo‐inositol and d‐chiro‐inositol in a 40:1 ratio, ALA, and *Gymnema sylvestre* were examined on glucose and lipid metabolic parameters	Two groups of 37 patients were established as follows: (i) the control group under a hypocaloric Mediterranean diet, and (ii) the study group received daily supplements consisting of two sachets containing 1950 mg myo‐inositol, 50 mg D‐chiro‐inositol, 50 mg ALA, and 250 mg *Gymnema Sylvestre* for 6 months	All metrics in control and treated groups improved following a 6‐month course of treatment. The treated group showed a larger improvement in terms of the deviation from the baseline in body weight, waist circumference, BMI, triglycerides, and insulin resistance. The results presented that these combined supplements are recommended as a therapeutic approach to enhance the positive effects of dietary programs in patients with dysmetabolism	Basciani et al. (2023)
Bovine serum albumin (BSA)					
*Akkermansia* *Allobaculum* *Alloprevotella*, *Dubosiella* *Lachnospiraceae* *Muribaculaceae*	20 α‐Klotho heterozygous hypomorphic male mice aged 6–8 weeks	The modifications in intestinal microbiota structure and immune system effects were examined in Klotho‐deficient mice under BSA treatment associated with kidney injury and chronic kidney disease	1 mg/g body weight BSA was administered to all mice by intraperitoneal gavage for the first 2 days after 1 week of adaptive feeding. Then, 1.5 mg/g of body weight BSA was administered by gavage for 3 weeks	BSA‐treated mice exhibited a considerably reduced relative abundance of the genera *Allobaculum* and *Muribaculaceae* and a significantly greater relative abundance of the genera *Dubosiella*, *Akkermansia, Alloprevotella*, and *Lachnospiraceae*. Abnormal protein expression in the Nrf2/NF‐κB signaling pathway in monocyte‐derived macrophage M1 cells exacerbates inflammation and kidney damage during immune activation and chronic inflammation caused by GM imbalance in Klotho‐deficient mice treated with BSA	Lai et al. (2021)
*Alistipes* sp. *Bacteroides ovatus* *Bacteroides vulgatus* *Escherichia coli, Lachnoclostridium edouardi* *Dorea longicatena, Coprococcus comes*	Four male and two female humans aged from 27 to 39 years old, on average 33.8 years old	The innovative ex vivo SIFR technique was used to examine the effects of three bovine plasma protein fractions on GM of six human adults	Serum‐derived immunoglobulins, plasma, and albumin‐enriched plasma were subjected to oral, gastric, and small intestinal digestion procedures in six human adults dosed up to 5 g/day	The limited range of gut microorganisms that serum‐derived bovine immunoglobulins regularly activated was very different from those that are normally engaged in the fermentation of carbohydrates. Protein bovine fractions could particularly alter the human GM to promote health benefits. The synthesis of SCFAs may result in the formation of a wider variety of metabolites generated from proteins. The prebiotics may be used to partially indigestible proteins in addition to ingestible carbohydrates	Van den Abbeele et al. (2023)
Human serum albumin					
*Aeromonas* *Alistipes, Alloprevotella* *Bacteroides* *Erysipelatoclostridium* *Escherichia* *Firmicutes* *Lactobacillus* *Parabacteroides* *Ruminiclostridium, Staphylococcus*	C57BL/6J male mice aged 8 weeks old with a weight of 20–22 g	Novel Se@Albumin complex nanoparticles, which were the self‐assembly of selenite salts with denatured human serum albumin, were examined to be used as a possible treatment for intestinal mucositis following chemotherapy by modulating GM	Mice were gavaged for 10 days with Se@Albumin complex nanoparticles with a dosage of 0.1 mg/kg/day. On the 7th day, mice received a single intraperitoneal injection of 10 mg/kg cisplatin	Se@Albumin complex nanoparticles could decrease intestinal mucositis in mice by restoring anti‐inflammatory microbes *Bacteroidetes* and *Firmicutes* and decreasing the amount of pro‐inflammatory bacteria *Escherichia*. Its activity may be directly mediated by GM modulation, as demonstrated by the findings that Se@Albumin NPs may significantly alleviate intestinal mucositis caused by cisplatin	Deng et al. (2021)
Lactoferrin (LF)					
*Acinetobacte* *Actinobacteria* *Bacteroides* *Butyricicoccus* *Candidatus* *Deferribacteres* *Desufobacterota* *Desulphurvibrio, Desulfovibrionia, Desulfovibrionales, Desulfovibrionaceae, Desulfovibrio* *Dubosiella* *Erysipelotrichales, Erysipelotrichaceae* *Fimicutes* *Ilebacterium valens* *Lachnospiraceae* *Lactobacillus* *Mucispirillum* *Oscillibacter* *Saccharimonas*	3‐week‐old male C57BL/6 mice	The possibility of using LF supplements in nutritionally obese was investigated in mice for the prevention and treatment of metabolic disorders in an effort to elucidate the mechanism of the intestinal microbes	21 mice were randomly divided into 3 groups: (1) control group (*n* = 7, 10% calories from fat), (2) high‐fat diet group (*n* = 7, 60% calories from fat), (3) high‐fat diet plus LF (*n* = 7, containing 2% LF) for 12 weeks with free access to water and diets. LF contained a purity of 95% and iron saturation of 15% and was dissolved 2 g/100 mL in distilled water	The visceral adipose ratio, blood glucose, triglycerides, total cholesterol, low‐density lipoprotein cholesterol, and *Firmicutes*/*Bacteroidetes* ratio were all significantly decreased in LF‐treated mice. The abundance of *Dubosiella* was considerably higher, whereas the abundance of *Deferribacteres*, *Oscillibacter*, *Butyricicoccus*, *Acinetobacter*, and *Mucispirillum* was much lower in the LF‐treated mice. GM's expression levels of genes involved in glucose metabolism rose, whereas those involving pyruvate metabolism reduced in the LF‐treated group. Through the regulation of gut flora, LF controls metabolic diseases	Yang et al. (2022)
*Alistipes* *Clostridium* XIVa, *Odoribacter*, *Ruminococcus*	5‐week‐old BALB/c male mice	The protective mechanism of LF and its impact on intestinal dysfunction in mice caused by deoxynivalenol was evaluated	24 mice were divided into 4 groups and treated as follows for 5 weeks: (1) peroral vehicle daily with commercial diet; (2) peroral 10 mg LF/day; (3) peroral 12 mg Deoxynivalenol/kg (4) peroral 10 mg LF/day and 12 mg Deoxynivalenol/kg	LF could significantly reduce levels of jejunal Il1b mRNA expression and phosphorylation of p38 and extracellular signal‐regulated kinase 1/2. Additionally, LF significantly increased colonic butyrate concentration and *Clostridium* XIVa relative abundance. The results demonstrated a novel anti‐mycotoxin strategy that uses LF to modify the gut microbial ecology and mitogen‐activated protein kinase pathway in mice, therefore alleviating intestinal dysfunction caused by deoxynivalenol	Hu et al. (2022)
*Actinobacillus* *Butyricimonas* *Collinsella* *Coprococcus* *Desulfovibrio* *Erysipelotrichaceae* *Escherichia–Shigella* *Howardella* *Lachnoclostridium* *Lactobacillus* *Prevotella 7* *Rikenellaceae RC9* *Roseburia* *Ruminococcus gauvreauii* , *Streptococcus* *Terrisporobacter* *Veillonella*	60 Duroc × Landrace × Yorkshire newborn piglets with an average weight of 1.51 ± 0.05 kg from six sows	The effects of an early‐life LF intervention on intestinal function, mucosal immunity, and colonic GM in nursing pigs were analyzed	On the first 7 days, the control group piglets were provided the same dosage of physiological saline as the LF group piglets received 0.5 g/kg body weight of LF solution daily. On days 8 and 21, six piglets from each of the two groups were selected at random and euthanized	LF‐treated piglets had higher colonic GM abundance‐based coverage estimator and Chao1 indices than those in the control group. Furthermore, the intestinal digesta of the LF‐treated piglets revealed a decreased quantity of *Escherichia‐Shigella* and a greater abundance of *Roseburia*. The colonic digesta of LF‐treated piglets also included more butyrate. On day 8, the LF‐treated piglets exhibited lower levels of Interleukin (IL)‐1α and IL‐1β in the colonic mucosa and a greater concentration of sIgA, but the LF‐treated piglets displayed an increase in IL‐10. These findings imply that early‐life LF intervention can enhance intestinal function in suckling pigs and modify the diversity of the colonic GM	Hu et al. (2020)
*Akkermansia* *Alistipes* *Bacteroides* *Firmicutes* *Helicobacter* *Intestinimona* *Lachnospiraceae* *Lactobacillus* *Muribaculaceae* NK4A136 group *Ruminococcaceae* UCG‐014 *Turicibacter* *Verrucomicrobia*	36 mice in colitis mouse model under induction of dextran sulfate sodium salt (DSSS)	The impact of bovine LF (BLF) on the intestinal barrier and inflammation was determined in mice under DSSS treatment, and the composition of intestinal flora and its metabolites in colitis was also demonstrated	Each of the 12 mice was randomly assigned to one of three groups. The control group regularly consumed water throughout the trial. In the DSSS treatment group, mice were fed regular saline for 14 days after they had freely consumed 4% DSSS solution for 7 days. After 7 days of unrestricted consumption of 4% DSSS solution, 100 mg/kg BLF was gavaged for 14 days in BLF‐treated mice	In the colon of BLF‐treated group, expressions of Claudin‐1, Occludin, TGF‐β, IL‐10, and ZO‐1 were higher; TNF‐α, IL‐6, and IL‐1β expressions were all lower. BLF‐treated mice had significantly altered alpha and beta diversity in 16S rDNA sequencing. BLF ameliorated colitis in mice by modulating the metabolites and composition of gut flora and improving the intestinal barrier's structure and inflammatory response	Wang et al. (2021)
*Acidobacteriota* *Alistipes* *Bacteroidota* *Bryobacter* *Firmicutes* *Psychrobacter*	30 male 3‐week‐old C57BL/6 mice with average weight 17 ± 1 g	The anti‐inflammatory and therapeutic benefits of LF in obese male mice were evaluated by examining intestinal flora	Thirty mice were randomly divided into 3 groups of 10 mice each: (1) the control group was fed a regular diet, (2) the high‐fat diet group, (3) after 2 weeks under a high‐fat diet, following 2% LF. All the mice were subjected to cervical dislocation under anesthesia after 12 weeks	LF improved intestinal barrier integrity by upregulating occludin protein and zonula occludens‐1 expression levels in the GI tract. By decreasing the ratio of *Firmicutes* and increasing the ratio of *Bacteroidota*, LF changed the intestinal microbial structure of obese mice. This resulted in a higher relative abundance of *Alistipes*, *Acidobacteriota*, *Psychrobacter*, and *Bryobacter* in the GI bacterial community	Wang, Zhang, et al. (2024)
*Bacteroides* *Blautia* *Bifidobacterium* *Collinsella* *Enterococcus* *Lactobacillus* *Megasphaera* *Peptoclostridium* *Prevotella* *Ruminococcaceae gnavus*	Six male and six female cats aged 2–3 months	LF and *Lactobacillus plantarum* ‐containing food supplements are examined for their impact on immune systems, digestive health, and GM composition in kittens	12 kittens were divided equally into two treatment groups of six kittens each. The control group with a regular diet and the experimental group with a regular diet included 500 mg/kg LF and 1 × 10^10^ CFU/kg *L. plantarum* CR12 under a 28‐day trial period	Ig A levels rose 14.9%, and IgG levels increased 14.2%, respectively, indicating that these supplements greatly improve immunological responses. The populations of *Lactobacillus* and other beneficial microbes grew from 4.13% to 79.03%, indicating a significant improvement in GI health. Pro‐inflammatory cytokine levels were also much lower in the treatment group, with drops of 13.94% in IL‐2, 26.46% in TNF‐α, and 19.45% in IFN‐γ. The potential of LF and *L. plantarum* as dietary therapies might enhance kitten health and reduce the need for antibiotics while minimizing related hazards	Dong et al. (2024)
*Akkermansia* *Allobaculum* *Bacteroides* *Clostridium* *Eisenbergiella* *Firmicutes* *Ileibacterium* *Intestinimonas* *Lactobacillus*	Eight‐week‐old C57BL/6J male mice	The fundamental mechanisms and the preventative benefits of LF were accessed in mice with alcoholic liver injury	Male mice were fed two distinct diets: the AIN‐93G diet for the ethanol and control groups and the AIN‐93G diet with 0.4% and 4% casein substituted by LF for the high‐dose and low‐dose LF groups for 8 weeks. Alcoholic liver injury is brought on by administering 20% ethanol ad libitum with multiple binges	LF reduced hepatic superoxide and inflammation levels, lessening hepatic damage by promoting the production of aldehyde dehydrogenase‐2 and inhibiting the overexpression of cytochrome P450 2E1. LF raises the relative abundances of *Lactobacillus* and *Akkermansia*. LF most likely acted in the form of its digestion products rather than the intact LF molecule under typical conditions	Li et al. (2021)
*Adlercreutzia, Allobaculum, Bifidobacterium* *Bilophila* *Clostridia* *Coprococcus* *Enterococcus* *Lachnospiraceae* *Lactobacillus* *Parabacteroides* *Ruminococcus*	Male C57BL/6J mice aged 10–12 weeks	A high‐fat, high‐cholesterol diet, including cholate, can cause nonalcoholic fatty liver disease. The current investigation aimed to discover whether LF intervention might prevent this condition in male mice	24 mice were randomly divided into 3 groups of 8 mice each: (1) the control group with a standard diet, (2) the high‐fat diet with 60% fat containing 1.25% cholesterol and 0.5% sodium cholate (3) the high‐fat diet with 1% LF. The mice were sacrificed after a 12 h fast following an 8‐week intervention	LF reduced the relative number of GM that promote 5‐hydroxytryptophan, such *Clostridia*, and increased the relative abundance of GM linked to glycolipid metabolism, including *Adlercreutzia*. LF raised SCFA levels, which correlated favorably with *Adlercreutzia* relative abundance. LF intervention suppressed nonalcoholic fatty liver disease in mice under A high‐fat, high‐cholesterol diet including cholate, which may be related to the composition of GM and the control of the HTR2A‐PPARa‐CPT‐1A pathway	Ding et al. (2025)
*Akkermansia* *Bacteroides* *Enterobacteriacea* *Enterococcus* *Lactobacillaceae* *Oscillospira* *Rikenellaceae* *Ruminococcaceae* *Streptococcus* *Veillonella* *Verrucomicrobiaceae*	Pediatric patients aged older than 1‐month receiving first‐line induction chemotherapy for acute lymphoblastic leukemia, acute myeloid leukemia and non‐Hodgkin's lymphoma	The ability of LF to reverse GMd brought on by chemotherapy was determined in pediatric patients	Pediatric patients with hematologic malignancies were recruited, and their GM was profiled by next‐generation 16S rRNA gene sequencing both before and after 2 weeks of oral supplementation with LF or placebo throughout induction chemotherapy	By promoting the preservation of variety and inhibiting the growth of pathobionts like *Enterococcus*, LF was safely provided without causing any negative side effects and promoting GM homeostasis while regulating the abundance of other taxa relevant to GI health like *Akkermansia*. Therefore, LF may be a viable addition to existing treatment approaches in these susceptible individuals to decrease the possibility of complications from GM	D'Amico et al. (2022)
*Acidaminococcascease* *Actinomycetaceae* *Bacteroides* *Bifidobacterium* *Brachyspiraceae* *Clostridia* *Enterococcus* *Lachnospiraceae* *Lactobacillus* *Ruminococcus* *Streptococcus* *Veillonella* *Verrucomicrobiaceae*	54 individuals with the median age 51 years old under viral suppression who were infected with the human immunodeficiency virus participated in a randomized, placebo‐controlled crossover clinical study of recombinant human LF	A pilot trial with recombinant human LF was assessed for its treatment safety, tolerability, and possible anti‐inflammatory effects	1500 mg LF twice day orally was compared to a placebo in a randomized, double‐blind, crossover clinical trial design to examine the therapy effects. The crossover strategy included a 2‐ to 4‐month washout phase in between two 3‐month treatment cycles. Period 1 included baseline, month 1, and month 3, while Period 2 included months 5, 6, and 8. Each period comprised three visits	No significant impact had been observed on intestinal microbiota diversity, mucosal integrity, monocyte/T‐cell activation, or plasma interleukin‐6 or D‐dimer levels. Although rh‐LF was safe for consumption orally, it had no effect on immune activation or inflammation	Sortino et al. (2019)
*Bifidobacterium*, *Holdemanella bioformis*	Female participants with 65–85 years old who had a BMI of 20–30, were in good mental health, non‐smoke, and had regular, typical Dutch eating habits were recruited	The impact on GI health based on intestinal inflammation and barrier indicators was estimated to describe the nutritional impacts of LF, galactooligosaccharides (GOS), and vitamin D on GM composition and function	25 senior women were divided into 2 groups at baseline (T1), each of whom received a dietary intervention (*n* = 12) or a placebo therapy (*n* = 13) for 9 weeks. The intervention group received 1 g/day BLF for the first 3 weeks of the trial, then 1 g/day BLF and 2.64 g/day active GOS for 3 weeks, and finally 1 g/day BLF, 2.64 g/day GOS, and 20 μg/day vitamin D for 3 weeks. Maltodextrin was given to the placebo group at the same doses as the intervention group	The intervention group's relative abundance of the species *Holdemanella* was significantly higher than that of the placebo. The relative abundance of *Bifidobacterium* in the intervention group was considerably greater than in the placebo group. By the end of the intervention, zonulin levels in the placebo group had considerably risen. The relative abundance of *Holdemanella* in the fecal microbiota of healthy older women was increased by supplementing with LF, while the relative abundance of *Bifidobacterium* was increased by supplementing with GOS	Konstanti et al. (2022)
*Acidobacteria* *Bacteroidetes* *Chloroflexi* *Euryarchaeota* *Firmicutes* NC10 *Nitrospirae*	12 men participants aged 18–65 years with healthy body weigh	The absorption and impact of Progel microencapsulated BLF on immunological indicators and GM were investigated	In a crossover, double‐blind, randomized 10‐week study, twelve good‐health male volunteers participated for two 4‐week supplementation arms with a 2‐week washout period. 200 and 600 mg of BLF were examined, both with and without Progel microencapsulation. Fasting blood tests were performed on all participants at the beginning, middle, and end of each trial period	After supplementation, the phylum‐level microbial community profile changed in the second trial arm, especially in those taking Progel microencapsulated LF. Additionally, it raised the levels of *Firmicutes* and *Bacteroidetes* while diminishing those of *Euryarchaeota*, *Acidobacteria*, *Chloroflexi*, NC10, and *Nitrospirae*. The results indicated that LF supplementation might enhance absorption and benefit the immune system and GM	Dix and Wright (2018)
*Bacillus* *Bacteroides* *Bifidobacterium* *Corynebacterium 1* *Clostridium sensu stricto 1* *Enterobacteriaceae* *Enterococcus* *Escherichia–Shigella* *Finegoldia* *Haemophilus* *Lactobacillus* *Staphylococcus* *Streptococcus* *Veillonella*	479 preterm infants with a mean gestation of 28.4 ± 2.3 weeks	The effects of LF on bacterial activity and GM were determined to identify any alterations that may occur before the beginning of illness	Trial participants were randomly assigned to receive either a blinded placebo sucrose or supplementary enteral BLF with 150 mg/kg/day from the time enteral milk feeds were tolerated until 34 weeks postmenstrual age	*Staphylococcus* demonstrated the most significant reduction in relative bacterial abundance over time, going from 42% at 7–9 days to just 2% at 30–60 days. LF considerably reduced the amount of *Staphylococcus* and other important infections, even though the effect was less pronounced than that of other clinical factors. Several inflammatory pathways that result in necrotizing enterocolitis were found by immunohistochemistry analysis	Embleton et al. (2021)
Lysozyme (LZ)					
*Atopobiaceae, Bacteroidetes, Bifidobacterium, Dubosiella, Firmicutes, Lactobacillua, Muribaculaceae*	35 male C57BL/6 mice aged 6–8 weeks old	The protection of LZ in intestinal stem cell niche was demonstrated in deoxynivalenol‐induced intestinal injury in male mice	35 mice were randomly divided into 5 groups of 7 mice each under 14‐day treatments. There are control group, dithizone (40 mg/kg) group, deoxynivalenol (2 mg/kg) group, dithizone + deoxynivalenol group, and LZ (200 U/day) + dithizone + deoxynivalenol group	The *Firmicutes/Bacteroidetes* ratio decreased following dithizone and deoxynivalenol treatments, and higher *Dubosiella* and lower *Lactobacillus* abundances were observed in mice. After the deoxynivalenol challenge, intestinal injury and intestinal stem cell loss in mice were reduced by the functional recovery of Paneth cells with 200 U/day LZ supplementation. Furthermore, intestinal organoids' growth and intestinal stem cell activity were enhanced by LZ	Cui et al. (2023)
*Actinobacteria* *Bacteroidota, Campilobacterota, Clostridium sensu stricto 1* *Firmicutes* *Olsenella* *Prevotella*	48 healthy weaned Landrace × Yorkshire piglets aged 22 days	The effects of LZ on the intestinal barrier and growth performance of weaned pigs were investigated in healthy weaned piglets	Piglets were divided into two groups at random by following dietary interventions. The investigation lasted 19 days and included two groups: (1) the control group (basal diet); and (2) LZ group (basal diet + 0.1% LZ)	LZ enhanced intestinal morphology and significantly elevated jejunal occludin expression. Furthermore, LZ suppressed the expression of genes involved in the nuclear factor‐k‐gene binding signaling pathway and downregulated the production of interleukin‐1β and tumor necrosis factor‐α. More significantly, LZ decreased the number of *Olsenella* and *Prevotella* at the genus level, increased the abundance of *Clostridium sensu stricto* 1, and the ratio of *Firmicutes* to *Bacteroidota* at the phylum level. LZ may successfully enhance weaned piglets' intestinal barrier function, reduce inflammation, and increase growth performance	Wu et al. (2023)
*Actinobacteria* *Bacillus* *Bacteroidetes* *Clostridium sensu stricto 1* *Eubacterium coprostanoligenes* *Escherichia‐Shigella* *Euryarchaeota* *Firmicutes* *Lactobacillus* *Methanobrevibacter* *Proteobacteria* *Ruminococcaceae NK4A214* *Ruminococcaceae UCG‐002* *Ruminococcaceae UCG‐005* *Spirochaetes* *Streptococcus* *Tenericute*	60 Yorkshire × Landrace sows with3–6 parity and 14.07 ± 2.58 mm backfat thickness	The impact of dietary LZ on sow metabolite variations and fecal microbial composition was investigated	60 sows included the control group (basal diet, *n* = 20), the LZ 150 diet group (basal diet +150 mg/kg LZ, *n* = 20), and the LZ 300 diet group (basal diet +300 mg/kg LZ, *n* = 20). The sows were fed a treatment diet from day 85 of gestation—typically farrowing occurs on day 114 of gestation—until the end of weaning on day 21 of lactation	The addition of LZ reduced SCFAs in the feces. With LZ supplementation, zonulin and endotoxin levels in the serum and feces were reduced. The addition of LZ altered the abundance of fecal microorganisms at various taxonomic levels. During lactation, there was a drop in *Bacteroidetes*, *Actinobacteria*, *Tenericutes*, and *Spirochaetes*, an increase in *Lactobacillus*, and a decrease in overall diversity. These results imply that dietary LZ supplementation from the end of gestation to lactation induces microbial modifications, which may be the mechanism by which the inflammatory state and maternal metabolites improved following LZ supplementation	Xu, Shi, et al. 2020; Xu, Curtasu, et al. 2020
Glycomacropeptide (GLMP)					
*Bacteroidaceae Bacteroides* *Christensenellaceae Catabacter* *Clostridiales vadinBB60* *Lachnospiraceae Anaerocolumna* *Lachnospiraceae ASF356* *Lachnospiraceae GCA‐900066575* *Lachnospiraceae Incertae Sedis* *Lachnospiraceae NK4A136 group* *Ruminococcacea, Butyricicoccus* *Ruminiclostridium 9* *Ruminococcaceae UCG‐014*	36 Eight‐week‐old C57BL/6 male mice	The protective influence of GLMP on metabolic syndrome was analyzed in male mice	36 mice were separated into 3 groups of 12 mice, each with three different dietary conditions for 12 weeks. Mice were orally fed either a standard chow with water only or a high‐fat, high‐fructose diet with 5.2 kcal/g containing 15% protein, 20% sucrose, 65% fat in addition to 30% fructose in drinking water. One group of mice also orally received a high‐fat, high‐fructose diet with 200 mg/kg GLMP	GLMP‐treated animals showed a tendency toward a reduction in bile acids without any evident alterations in the composition of their intestinal flora. Given its positive effects on risk variables, including inflammation, oxidative stress, and endoplasmic reticulum stress, without affecting GM, GLMP provides significant promise for combating metabolic syndrome‐related components and effects	Sauvé et al. (2024)
*Bifidobacterium* *Enterococcus faecium* *E. coli* *Klebsiella aerogenes* *Klebsiella pneumoniae*	Very preterm healthy infants who were born between 28 and 32 weeks gestation and whose mothers were unable to provide enough breast milk or chose not to breastfeed were recruited	The composition and function of GM of very preterm newborns were evaluated with the effects of an infant formula that contained the particular prebiotic combination of the ratio of short‐chain GOS and *long‐chain fructo‐oligosaccharides* (9:1) and GLMP	GM of very preterm infants was obtained four times to undertake metagenomic analysis: 24 h before the trial and 7, 14, and 28 days following the study. The experimental formula contained a prebiotic mixture providing 0.65 g the ratio of short‐chain GOS and *long‐chain fructo‐oligosaccharides* (9:1) and casein GLMP providing 40 mg sialic acid/100 mL	The major species in the treatment group and the control group were *Klebsiella aerogenes* , *E. coli, Enterococcus fecium*, and *Klebsiella pneumoniae* . The treatment group's *Bifidobacterium* abundance dramatically increased following the 4‐week intervention. The findings indicated that the intestinal microecology of very preterm newborns benefits from formula enhanced with the ratio of short‐chain GOS and ***long‐chain** fructo‐oligosaccharides* (9:1) and GLMP	Yu et al. (2022)
*Agathobacter Lachnospiraceae* *Streptococcus*	13 Eligible women had no underlying inflammatory conditions, a BMI of 28 to 35 kg/m^2^, and were at least 10 years postmenopausal with age younger than 90 years old	The effects of GLMP on satiety, glucose homeostasis, inflammation, and the fecal microbiota were assessed in 13 obese women in a dose‐finding crossover study for 14 days	13 women with mean aged 57 years old, a median of 8 years (range from 3 to 9 years) past menopause and a mean BMI of 30. GLMP supplements (15 g GLMP +10 g WPs) were taken twice daily for 1 week and three times daily for another week, with a washout period in between. On the first day of each week with soy and the seventh day with GLMP, women took a meal tolerance test	GLMP substantially increased the amylin concentration of the postprandial area under the curve compared to soy meal tolerance test, and it was associated with higher satiety and C‐peptide. Consuming GLMP supplements three times a day decreased entire α diversity, while consuming them twice a day decreased members of the genus *Streptococcus*	Hansen et al. (2023)
*Agathobacter* *Akkermansia* *Alistipes* *Bacteroides* *Blautia* *Dialister* *Faecalibacterium, Lachnospiraceae* *Parabacteroides* *Ruminococcus 2* *Subdoligranulum*	9 Pediatric Patients with annual mean phenylalanine levels within the range ofd 120–360 μmol/L in childhood (6–12 years old) and 120–600 μmol/L in adolescence and adult age (> 12 years old)	The impact of GLMP supplementation on GM of nine Phenylketonuria patients was evaluated by comparing GM before and after the 6‐month intervention under 16S rRNA sequencing analysis	The GLMP intervention, which replaced the AAs with GLMP, began following the baseline visit and lasted < 4 weeks. In the group under 6 months after GLMP ingestion, at least 30% of the AA supplementation from the GLMP formulation had been reached	The particular prebiotic impact on the butyrate‐producing *Agathobacter* spp. and lesser influence on *Subdoligranulum* was found, but no significant alterations in GM were observed. The findings point to GLMP as a safe substitute in the PKU diet and its potential prebiotic function on some taxa without significantly altering the commensal GM	Montanari et al. (2022)
*E. coli* *Salmonella typhimurium* *Shigella flexneri* *Streptococcus mutans*	34 healthy Caucasians aged between 24 and 59 years, BMI of 18–25 kg/m^2^ and absence of lactose intolerance, milk protein allergy and chronic disease	The systemic and intestinal immunomodulatory effects of casein GLMP taken orally were evaluated in 34 healthy Caucasians	Participants was under a 4‐week intervention of either 25 g of oral powder‐based chocolate‐flavored casein GLMP or a reference drink in a single‐centre limited but randomized, double‐blinded, reference‐controlled study	Comparing casein GLMP to a reference drink, it demonstrated no systemic or intestinal immunomodulatory effects on fecal calprotectin or the high‐sensitivity C‐reactive protein levels, fecal microbiota composition, or fecal SCFAs content. There were no serious side effects from asein GLMP consumption, and it had no effect on body weight or satiety. There was no modification or induction of GI symptoms by casein GLMP	Wernlund et al. (2021)

### 3.1 ß‐Lactoglobulin

BLG exhibits several interesting activities on GM through direct and indirect mechanisms. The 3D structure and bioactivity of this protein contribute significantly to the net impact of whey on microbial communities of the intestinal tract. As the major WP constituent, BLG is found in the colon as only partially hydrolyzed due to its comparatively stable globular structure that resists complete hydrolysis by human digestive enzymes. This structural resistance allows it to serve as a substrate for lower GI proteolytic bacteria. Upon metabolism by these microbes, BLG yields characteristic peptide fragments with varying effects on different bacterial populations (Barone et al. 2020). Various BLG‐derived peptides possess antimicrobial activity that selectively kill pathogenic bacteria with minimal effect on commensal strains. For example, BLG sequence fragments have inhibited *Escherichia coli* and certain *Clostridium* species via mechanisms including destruction of bacterial cell membranes, and so forth. This selective action impacts microbial community structure by suppressing potential pathogens.

BLG‐derived peptides also function as signaling molecules modulating bacterial gene expression and metabolism. Some peptides have been reported to modulate quorum sensing systems, used by bacteria to coordinate behavior as a function of population density (Lee et al. 2024). This modulation controls biofilm development, virulence factor production, and interspecies communication in GM, potentially favoring more symbiotic community architecture (Chen et al. 2022). The protein structure consists of specific regions that, on release during bacterial catabolism, are capable of acting as growth factors for beneficial bacteria such as *Bifidobacterium* and *Lactobacillus* species. The growth stimulation does appear to be strain‐specific, with some of the beneficial bacteria possessing the enzymic equipment to utilize BLG‐derived peptides as preferred sources of nitrogen. This particular growth advantage results in increased populations of these beneficial genera following WPs consumption (Bamdad et al. 2017; Lee et al. 2025).

It does so by its binding function, influencing the availability of other nutrients to GM. BLG binds retinol, fatty acids, and other hydrophobic molecules, potentially altering their delivery to diverse microbial populations and the influence on metabolic pathways dependent on these nutrients. This carrier function is an indirect mechanism through which BLG regulates microbial community structure and function. Recent work has also indicated that BLG can influence the intestinal mucus layer, in which most GM species reside. Through interaction with mucin glycoproteins, BLG can modify the adhesion properties of the mucus, stimulating colonization by certain bacterial species and repressing others. This action through the mucus is yet another way in which BLG controls the spatial organization of GM along the intestine (Cronin et al. 2018; Master and Macedo 2021).

A mouse model of food allergy was used to investigate if *Clostridium butyricum* CGMCC0313‐1 may alleviate intestine anaphylaxis caused by BLG. The results displayed that *Clostridium butyricum* significantly decreased the symptoms of intestinal anaphylaxis by enhancing Secretory IgA and CD4+ CD25+ Foxp3Treg cells. The imbalance in the expression of Th1/Th2 and Th17/Treg transcription factors was also rectified (Zhang et al. 2017). The relationship between Th17‐dominated BLG allergy prevention and *Lactobacillus acidophilus* capacity to regulate miRNA expression was investigated in an allergic mouse model. Live *L. acidophilus* treatment significantly inhibited Th17 proliferation and hypersensitivity reactions. Furthermore, live *L. acidophilus* decreased the expression of four miRNAs, namely miR‐146a and miR‐155. The reduction in Interleukin (IL)‐17 and RORγt mRNA expression was directly linked to the reduced expression of miRNAs in the group treated with *L. acidophilus* (Wang et al. 2018).

The identification of new biomarkers and the development of creative preventative and therapeutic approaches depended on an understanding of the fundamental mechanisms underlying IgE‐mediated cow's milk allergy. The metabolic changes that underlie IgE‐mediated cow's milk allergy in children were determined. Several metabolic changes were caused by microbes during mouse sensitization, particularly in the metabolites of bile acid, energy, and tryptophan, which came before allergic inflammation. Using in vitro digestions and multi‐omics approaches, the study demonstrated microbial dysbiosis and its subsequent effects on metabolic changes in children with cow's milk allergy. This was accompanied by biochemical indicators of low‐grade inflammation. The findings suggested that GMd in children following an elimination diet stimulated a persistent low‐grade inflammation that preceded allergic inflammation (De Paepe et al. 2024).

### 3.2 α‐Lactalbumin

ALA, the second most abundant protein of bovine whey and the predominant WP in human milk, has significant impacts on microbial gut metabolic activity and on microbial community structure through a number of novel mechanisms. As a calcium‐binding metalloprotein of dense globular conformation, ALA is highly resistant to gastric digestion, allowing large quantities to reach the lower GI tract in bioactive form (Boscaini et al. 2019). Its structural integrity allows direct engagement with colonic GM, where ALA is both a substrate and modulator of bacterial metabolism. Compared to BLG, the molecular topology of ALA presents hydrophilic areas to increased access by bacterial proteases, resulting in different patterns of microbial utilization (Boscaini et al. 2021; Chen et al. 2020).

ALA was discovered to specifically stimulate the growth of *Bifidobacterium* species that are beneficial for health, including *B. longum* and *B. breve* , by using specific oligosaccharide‐binding sites that adhere to the surface of bacteria. Such adhesions trigger the proliferation and metabolic processes of such beneficial bacteria, which further lead to the production of SCFAs via fermentation. Acidic environments are created that selectively promote acid‐tolerant commensal species and suppress potential pathogens, creating a positive feedback process that strengthens community structure changes to favor host health (Chen et al. 2021). The impact of *Lactiplantibacillus plantarum* HM‐22 on intestinal inflammation and intestinal microbiota was examined in mice with ALA‐induced allergies. *L. plantarum* raised levels of IL‐10, interferon‐γ, and transforming growth factor‐β while lowering levels of total IgE and the proinflammatory factor IL‐4. By using *L. plantarum* HM‐22, the intestinal microbes of ALA‐induced allergic mice improved, the crypt structure of their colon tissues changed, goblet cells decreased, and the phenomenon of numerous inflammatory corpuscles appeared was improved and alleviated. So, this probiotic *L. plantarum* HM‐22 can prevent allergies by altering the gut flora (Jiang et al. 2021).

The effects of varying ALA concentrations on the variety and structure of gut flora and the optimal concentration of ALA adding to infant formula were determined under the male rat model. SD rats were divided into four groups and received 200, 100, and 20 mg/kg of ALA and an equivalent amount of normal saline as a control group orally. After 14 and 28 days, colonic feces were aseptically removed. The findings indicated that the species richness, diversity, and structure of intestinal microbiota were enhanced by 200 and 100 mg/kg of ALA, with an impact comparable to that of breast milk. The relative abundance of *Lactobacillus*, *Blautia*, and *Akkermansia* was decreased by high ALA. All concentrations of ALA maintained the relative abundance of *Ruminococcaceae*, *Clostridium sensu stricto* 1, and *Lachnospiraceae* species at the same level while decreasing the relative abundance of *Prevotella* and *Bacteroides* and increasing the relative abundance of *Eubacterium xylanophilum* . It was suggested that the medium dose of 100 mg/kg of ALA was optimal for controlling intestinal microbiota since it improved intestinal microbial diversity, maintained or raised the relative abundance of intestinal probiotics, and decreased the levels of intestinal pathogenic bacteria (Menghan et al. 2020).

Supplementing with WPC enriched with ALA may enhance the development of newborn pigs. Dilute bovine milk, supplemented with WPC with normal or high levels of ALA, was administered to 40 preterm piglets delivered by cesarean section. High levels of ALA tended to exhibit a higher growth rate, a quicker meal transit time, increased bone mineral content, more colon microbial diversity, and a higher abundance of certain bacteria and microbial metabolites. WPC supplementation in milk could enhance relative organ weights, blood AAs, blood neutrophil function, and microbial metabolites (Nielsen et al. 2020). The capacity of ALA was accessed on the effects in treating polycystic ovary syndrome (POS) to restore GMd. ALA may have a role in increasing the growth of *Bifidobacterium* and *Lactobacillus* that promote health while restricting the growth of possible pathogens, according to studies performed on women with POS who received oral ALA for 30 days. The findings may help restrict or repair intestinal dysbiosis associated with POS by establishing ALA as a legitimate substance with prebiotic effects (Alessandri et al. 2024; Cardinale et al. 2022).

The effects of a hypocaloric Mediterranean diet combined with supplements of myo‐inositol and d‐chiro‐inositol in a 40:1 ratio, ALA, and *Gymnema sylvestre* were examined on glucose and lipid metabolic parameters. All metrics in control and treated groups improved following a 6‐month course of treatment. Nonetheless, the treated group showed a larger improvement, particularly in terms of the deviation from the baseline in body weight, waist circumference, body mass index, triglycerides, and insulin resistance. The results presented that these combined supplements are recommended as a therapeutic approach to enhance the positive effects of dietary programs in patients with dysmetabolism (Basciani et al. 2023).

The peptide VGINYW and ALA hydrolysates under 3 kDa were evaluated for their antihypertensive impact, and the potential mechanism was demonstrated in spontaneously hypertensive rats administered with 5 mg/kg VGINYW and 100 mg/kg ALA hydrolysates. In addition to significantly lowering the systolic blood pressure of the SHRs, VGINYW and ALA hydrolysates may also significantly reduce oxidative stress within the spontaneously hypertensive rats. More importantly, by restoring the diversity of GM and modulating the critical floras that produce SCFAs, the gavage of VGINYW and ALA hydrolysates could potentially treat intestinal microbiota dysbiosis linked to hypertension (Xie et al. 2022). The same research team used the isolated GI hydrolysates of ALA to examine the possible anti‐hyperuricemic and nephroprotective effects on hyperuricemic mice. The mice were administered 300 mg/kg potassium oxonate and 300 mg/kg hypoxanthine orally every day to induce hyperuricemia. The results demonstrated that the levels of serum uric acid, creatinine, and urea nitrogen were significantly reduced. GM assessments showed that ALA hydrolysate treatment suppressed the proliferation of genera linked to inflammation and hyperuricemia while increasing the abundance of certain producers of mice fecal SCFAs. By reducing oxidative stress and inflammation and modifying GM in hyperuricemic rats, the isolated GI hydrolysates of ALA exhibited anti‐hyperuricemia and nephroprotective effects (Xie et al. 2023).

### 3.3 Serum Albumins (Bovine/Human)

Serum albumin, making up 5%–10% of WPs, has certain actions on microbial gut metabolism and community structure through mechanisms very distinct from other WP constituents. As the most abundant protein found in plasma blood that is eventually secreted into milk, the unique molecular properties of serum albumin enable it to have important effects on the intestinal microbiome (Ghafoori et al. 2022; Zhang et al. 2021). Serum albumin possesses superior binding capacity for small molecules like fatty acids, hormones, and xenobiotics. Binding capacity in turn largely regulates microbial metabolism by changing the bioavailability of such compounds in the gut environment. When partially digested albumin reaches the colon, it still carries bound substrates that can be mobilized by bacterial metabolism, creating microenvironments with different substrate availability and thus preferring some microbial species over others. For instance, released fatty acids during the bacterial breakdown of albumin are carbon substrates that *Faecalibacterium* and *Roseburia* species preferentially utilize (Farooq et al. 2019).

Proteolytic digestion of serum albumin by intestinal bacteria liberates BAPs with selective activity against microbial populations. Several BSA‐derived peptides have been discovered to possess selective antimicrobial activity against pathogenic bacteria like *Salmonella typhimurium* and enteropathogenic *E. coli* but little inhibitory activity against health‐enhancing commensals (Lee et al. 2016). These peptides typically act through mechanisms of membrane disruption that exploit compositional differences in bacterial membranes between commensal and disease‐causing species, thereby organizing the community by selective pressure against potential pathogens. Serum albumin's impacts extend to microbial metabolism into its role in affecting bacterial enzymatic functions. BSA has the ability to control the action of bacterial β‐glucuronidase, a xenobiotic metabolizing enzyme and one associated with toxic metabolite formation. By preventing the action of this enzyme in certain bacterial genera, albumin reduces the formation of potentially toxic substances and brings about a condition favorable to bacteria with alternative means of metabolism, shifting community composition toward a possibly more benign character (Bergia et al. 2018; Bielecka et al. 2022).

The modifications in intestinal microbiota structure and immune system effects were examined in Klotho‐deficient mice under BSA treatment associated with kidney injury and chronic kidney disease. Compared to wild‐type mice, BSA‐treated mice exhibited a considerably reduced relative abundance of the genera *Allobaculum* and *Muribaculaceae* and a significantly greater relative abundance of the genera *Dubosiella*, *Akkermansia, Alloprevotella*, and *Lachnospiraceae*. Abnormal protein expression in the Nrf2/NF‐κB signaling pathway in monocyte‐derived macrophage M1 cells exacerbates inflammation and kidney damage during immune activation and chronic inflammation caused by GM imbalance in Klotho‐deficient mice treated with BSA (Lai et al. 2021; Lee 2023b). The innovative ex vivo SIFR technique was used to examine the effects of three bovine plasma protein fractions (serum‐derived Igs, plasma, and albumin‐enriched plasma) on GM of six human adults. The limited range of gut microorganisms that serum‐derived bovine Igs activated was very different from those typically engaged in the fermentation of carbohydrates. The serum‐derived bovine Igs‐fermenting community comprised the butyrate‐producing bacterium SS3/4, *Dorea longicatena*, *Coprococcus comes* , and *Bacteroides vulgatus* and *Lachnoclostridium edouardi* correlating with acetate and propionate. The results confirmed that protein bovine fractions could particularly alter the human GM to promote health benefits. Although the synthesis of SCFA may provide health benefits, it may also result in the formation of a wider variety of metabolites generated from proteins.

The prebiotics may be used to partially indigestible proteins and ingestible carbohydrates (Van den Abbeele et al. 2023). Novel Se@Albumin complex nanoparticles (NPs), which were the self‐assembly of selenite salts with denatured human serum albumin, were examined as a possible treatment for intestinal mucositis following chemotherapy by modulating GM. Se@Albumin complex NPs could decrease intestinal mucositis in mice by restoring anti‐inflammatory microbes *Bacteroidetes* and *Firmicutes*, and decreasing the amount of proinflammatory bacteria *Escherichia*. Its activity may be directly mediated by GM modulation, as demonstrated by the findings that Se@Albumin NPs may significantly alleviate intestinal mucositis caused by cisplatin (Deng et al. 2021; Lee 2025b).

### 3.4 Lactoferrin

LF, a bioactive glycoprotein in WPs, has a significant influence on the gut microbial population structure and microbial metabolism. It not only influences the type of microorganisms that colonize the gut but also the nature of the metabolic processes they carry out. All these effects are mainly due to LF's antimicrobial properties, iron‐binding affinity, and interaction with the host immune system (Abad et al. 2021). One of the most significant ways LF acts to modify gut microbial community structure is by selectively inhibiting the growth of pathogenic microorganisms. It sequesters free iron in a tight‐binding manner, making it unavailable to most microbes, hence starving iron‐requiring pathogens such as *E. coli* , *Salmonella*, and *Clostridium difficile* . In addition to iron deprivation, LF and its derivative peptides, such as lactoferricin, can kill bacteria directly by disrupting their membranes, particularly those of Gram‐negative organisms (Lee 2023a). Consequently, the relative concentration of pathogenic microbes in the gut is lower. Conversely, LF promotes the growth of advantageous bacteria, i.e., *Lactobacillus* and *Bifidobacterium*. These commensal organisms use a minimal amount of iron and can persist in the LF‐conditioned environment. Favorable promotion of advantageous microbes promotes more microbial diversity and harmony in the gut. More diversity and harmony in GM are associated with improved gut health, immunity against infection, and reduced inflammation (Hazra et al. 2021; Zandona et al. 2021).

The possibility of using LF supplements in nutritionally obese mice was investigated for the prevention and treatment of metabolic disorders in an effort to elucidate the mechanism of the intestinal microbes. The visceral adipose ratio, blood glucose, triglycerides, total cholesterol, low‐density lipoprotein cholesterol, and *Firmicutes*/*Bacteroidetes* ratio significantly decreased in LF‐treated mice. The abundance of *Dubosiella* was considerably higher, whereas the abundance of *Deferribacteres*, *Oscillibacter*, *Butyricicoccus*, *Acinetobacter*, and *Mucispirillum* was much lower in the LF‐treated mice. GM's expression levels of genes involved in glucose metabolism rose, whereas those involving pyruvate metabolism reduced in the LF‐treated group. By regulating gut flora, LF controls metabolic diseases (Yang et al. 2022). The protective mechanism of LF and its impact on intestinal dysfunction in mice caused by deoxynivalenol was evaluated. LF could significantly reduce levels of jejunal Il1b mRNA expression and phosphorylation of p38 and extracellular signal‐regulated kinase 1/2. Additionally, LF significantly increased colonic butyrate concentration and *Clostridium* XIVa relative abundance. The results demonstrated a novel anti‐mycotoxin strategy that uses LF to modify the gut microbial ecology and mitogen‐activated protein kinase pathway in mice, therefore alleviating intestinal dysfunction caused by deoxynivalenol (Hu et al. 2022).

The effects of an early‐life LF intervention on intestinal function, mucosal immunity, and colonic microbiota in nursing pigs were analyzed. On the first 7 days, the control group piglets were provided the same dosage of physiological saline as the LF group piglets received 0.5 g/kg body weight of LF solution daily. On days 8 and 21, six piglets from each of the two groups were selected at random and euthanized. LF‐treated piglets had higher colonic GM abundance‐based coverage estimator and Chao1 indices than those in the control group. Furthermore, the intestinal digesta of the LF‐treated piglets revealed a decreased quantity of *Escherichia‐Shigella* and a greater abundance of *Roseburia*. The colonic digesta of LF‐treated piglets also included more butyrate. On day 8, the LF‐treated piglets exhibited lower levels of IL‐1α and IL‐1β in the colonic mucosa and a greater concentration of sIgA, but the LF‐treated piglets displayed an increase in IL‐10. These findings imply that early‐life LF intervention can enhance intestinal function in suckling pigs and modify the diversity of the colonic GM (Hu et al. 2020; Liorančas and Lee 2024b).

The absorption and impact of Progel encapsulated bovine LF (BLF) on immunological indicators and GM were investigated. In a crossover, double‐blind, randomized 10‐week study, 12 male volunteers in good health participated; 200 and 600 mg of BLF were examined, both with and without Progel encapsulation (InferrinTM). Fasting blood tests were performed on all participants at the beginning, middle, and end of each trial period. After supplementation, the phylum‐level microbial community profile changed in the second trial arm, especially in those taking Progel encapsulated LF. Additionally, it raised the levels of *Firmicutes* and *Bacteroidetes* while diminishing those of *Euryarchaeota*, *Acidobacteria*, *Chloroflexi*, NC10, and *Nitrospirae*. The results indicated that LF supplementation might enhance absorption and benefit the immune system and GM (Dix and Wright 2018). The effects of LF on bacterial activity and GM were determined to identify any alterations that may occur before the beginning of illness. For this, 479 preterm infants were randomly assigned to receive either blinded placebo sucrose or supplementary enteral BLF with 150 mg/kg/day from the time enteral milk feeds were tolerated until 34 weeks postmenstrual age. The results exhibited that *Staphylococcus* demonstrated the most significant reduction in relative bacterial abundance over time, going from 42% at 7–9 days to just 2% at 30–60 days. The placebo group had higher mean relative abundances of *Staphylococcus*, *Haemophilus*, and *Lactobacillus* than the LF group. LF considerably reduced the amount of *Staphylococcus* and other important infections, even though the effect was less pronounced than other clinical factors. Several inflammatory pathways that result in necrotizing enterocolitis were found by immunohistochemistry analysis (Embleton et al. 2021).

The impact of BLF on the intestinal barrier and inflammation was determined in mice under the treatment of dextran sulfate sodium salt (DSSS), and the composition of intestinal flora and its metabolites in colitis was also demonstrated. Twelve mice each were randomly assigned to one of three groups. The control group regularly consumed water throughout the trial. In the DSSS treatment group, mice were fed regular saline for 14 days after they had freely consumed 4% DSSS solution for 7 days. After 7 days of unrestricted consumption of 4% DSSS solution, 100 mg/kg BLF was gavaged for 14 days in BLF‐treated mice. In the colon of the BLF‐treated group, expressions of Claudin‐1, Occludin, TGF‐β, IL‐10, and ZO‐1 were higher; TNF‐α, IL‐6, and IL‐1β expressions were all lower. BLF‐treated mice had significantly altered alpha and beta diversity in 16S rDNA sequencing. BLF ameliorated colitis in mice by modulating the metabolites and composition of gut flora and improving the intestinal barrier's structure and inflammatory response (Wang et al. 2021). The anti‐inflammatory and therapeutic benefits of LF in obese male mice were evaluated by examining intestinal flora. LF improved intestinal barrier integrity by upregulating occludin protein and zonula occludens‐1 expression levels in the GI tract. By decreasing the ratio of *Firmicutes* and increasing the ratio of *Bacteroidota*, LF changed the intestinal microbial structure of obese mice. This resulted in a higher relative abundance of *Alistipes*, *Acidobacteriota*, *Psychrobacter*, and *Bryobacter* in the GI bacterial community (Wang, Zhang, et al. 2024).

LF and *Lactobacillus plantarum* ‐containing food supplements are examined for their impact on immune systems, digestive health, and GM composition in kittens. IgA levels rose 14.9%, and IgG levels increased 14.2%, respectively, indicating that these supplements greatly improve immunological responses. The populations of *Lactobacillus* and other beneficial microbes grew from 4.13% to 79.03%, indicating a significant improvement in GI health. Proinflammatory cytokine levels were also much lower in the treatment group, with drops of 13.94% in IL‐2, 26.46% in TNF‐α, and 19.45% in IFN‐γ. These results highlight the potential of LF and *Lactobacillus plantarum* as dietary therapies that can enhance kitten health and reduce the need for antibiotics while minimizing related hazards (Dong et al. 2024). The fundamental mechanisms and the preventative benefits of LF were assessed in mice with alcoholic liver injury. LF reduced hepatic superoxide and inflammation levels, which in turn lessens hepatic damage by promoting the production of aldehyde dehydrogenase‐2 and inhibiting the overexpression of cytochrome P450 2E1. LF raises the relative abundances of *Lactobacillus* and *Akkermansia*. LF most likely acted in the form of its digestion products rather than the intact LF molecule under typical conditions (Li et al. 2021).

A high‐fat, high‐cholesterol diet, including cholate can cause non‐alcoholic fatty liver disease. The current investigation aimed to discover whether LF intervention might prevent this condition in male mice. LF reduced the relative number of GM that promote 5‐hydroxytryptophan, such as *Clostridia*, and increased the relative abundance of GM linked to glycolipid metabolism, including *Adlercreutzia*. Additionally, LF raised SCFA levels, which had a favorable correlation with *Adlercreutzia* relative abundance. LF intervention suppressed non‐alcoholic fatty liver disease in mice under a high‐fat, high‐cholesterol diet including cholate, which may be related to the composition of GM and the control of the HTR2A‐PPARa‐CPT‐1A pathway (Ding et al. 2025). The ability of LF to reverse GMd brought on by chemotherapy was determined in pediatric patients aged older than 1 month. Pediatric patients with hematologic malignancies were recruited, and their GM was profiled by next‐generation 16S rRNA gene sequencing both before and after 2 weeks of oral supplementation with LF or placebo throughout induction chemotherapy. By promoting the preservation of variety and inhibiting the growth of pathobionts like *Enterococcus*, LF was safely provided without causing any negative side effects and promoting GM homeostasis while regulating the abundance of other taxa relevant to GI health like Akkermansia. Therefore, LF may be a viable addition to existing treatment approaches in these susceptible individuals to decrease the possibility of complications from GM (D'Amico et al. 2022).

A pilot trial with recombinant human LF was assessed for its treatment safety, tolerability, and possible anti‐inflammatory effects; 54 individuals with a median age of 51 years old under viral suppression who were infected with the human immunodeficiency virus participated in a randomized, placebo‐controlled crossover clinical study of recombinant human LF. Persons with human immunodeficiency virus aged ≥ 40 years undergoing continuous effective antiretroviral therapy and human immunodeficiency virus RNA levels < 200 copies/mL for ≥ 1 year before enrollment were eligibility requirements. No significant impact was observed on intestinal microbiota diversity, mucosal integrity, monocyte/T‐cell activation, or plasma IL‐6 or D‐dimer levels. Although rh‐LF was safe for consumption orally, it had no effect on immune activation or inflammation (Sortino et al. 2019). The impact on GI health based on intestinal inflammation and barrier indicators was estimated in healthy elderly women to describe the nutritional impacts of LF, galactooligosaccharides (GOS), and vitamin D on GM composition and function. The intervention group's relative abundance of the species *Holdemanella* was significantly higher than that of the placebo. The relative abundance of *Bifidobacterium* in the intervention group was considerably greater than in the placebo group. By the end of the intervention, zonulin levels in the placebo group had considerably risen. The relative abundance of *Holdemanella* in the fecal microbiota of healthy older women was increased by supplementing with LF, while the relative abundance of *Bifidobacterium* was increased by supplementing with GOS (Konstanti et al. 2022).

### 3.5 Lysozyme

LZ, WP's antimicrobial enzyme, plays important functions in modulating GM by influencing the community structure and GM's metabolic activity. Its main mode of action is enzymatic—dissolving the peptidoglycan layer of bacterial cell walls, especially those of Gram‐positive bacteria—leading to lysis and death of certain microbes. Its action has ripple effects on microbial diversity, composition, and metabolic function in the gut (Aslam et al. 2020; Liorančas and Lee 2024a). At the level of community structure, LZ disproportionately reduces the counts of specific groups of bacteria, particularly Gram‐positive infections such as *Clostridium* and *Staphylococcus* species. By reducing these counts, LZ helps to reduce infection and inflammation risk in the gut. In contrast, competitively superior commensal and probiotic genera, e.g., *Bifidobacterium* and *Lactobacillus*, are less affected or even thrive in this setting, especially when competitive pathogenic pressure is blocked. This selective pressure can lead to a healthier and more balanced microbial community (Beam et al. 2021).

The action mechanism of LZ on microbial metabolism is closely connected with these structural changes. Through the inhibition of proteolytic and pathogenic bacteria, there is a shift in microbial metabolism away from the production of harmful metabolites such as ammonia, phenols, and hydrogen sulfide—byproducts associated with GMd and inflammation. Alternatively, beneficial bacteria may induce increased production of SCFAs, including acetate, propionate, and butyrate. Such metabolites preserve intestinal barrier function, modulate immune responses, and provide energy to the cells in the intestines (Minj and Anand 2020; Pillai et al. 2024). The protection of LZ in the intestinal stem cell niche was demonstrated in deoxynivalenol‐induced intestinal injury in male mice. The *Firmicutes/Bacteroidetes* ratio decreased following dithizone and deoxynivalenol treatments, and higher *Dubosiella* and lower *Lactobacillus* abundances were observed in mice. After the deoxynivalenol challenge, intestinal injury and intestinal stem cell loss in mice were reduced by the functional recovery of Paneth cells with 200 U/day LZ supplementation. Furthermore, intestinal organoids' growth and intestinal stem cell activity were enhanced by LZ (Cui et al. 2023).

The effects of LZ on the intestinal barrier and growth performance of weaned pigs were investigated in 48 healthy weaned piglets aged 22 days. LZ enhanced intestinal morphology and significantly elevated jejunal occludin expression. Furthermore, LZ suppressed the expression of genes involved in the nuclear factor‐k‐gene binding signaling pathway and downregulated the production of IL‐1β and tumor necrosis factor‐α. More significantly, LZ decreased the number of *Olsenella* and *Prevotella* at the genus level, increased the abundance of *Clostridium sensu stricto 1*, and the ratio of *Firmicutes* to *Bacteroidota* at the phylum level. LZ may successfully enhance weaned piglets' intestinal barrier function, reduce inflammation, and increase growth performance (Wu et al. 2023). The impact of dietary LZ on sow metabolite variations and fecal microbial composition was investigated. SCFAs in the feces were reduced by the addition of LZ. With LZ supplementation, zonulin and endotoxin levels in the serum and feces were reduced. The addition of LZ altered the abundance of fecal microorganisms at various taxonomic levels. During lactation, there was a drop in *Bacteroidetes*, *Actinobacteria*, *Tenericutes*, and *Spirochaetes*, an increase in *Lactobacillus*, and a decrease in overall diversity. These results imply that dietary LZ supplementation from the end of gestation to lactation induces microbial modifications, which may be the mechanism by which the inflammatory state and maternal metabolites improve following LZ supplementation (Xu, Shi, et al. 2020; Xu, Curtasu, et al. 2020).

### 3.6 Glycomacropeptide

GLMP, a bioactive WP peptide, has important effects on microbial metabolism and GM structure. GLMP is extremely rich in sialic acid and branched‐chain carbohydrates and hence acts as a functional food component with prebiotic functions. Due to these characteristics, GLMP selectively regulates GM and modulates microbial‐originated metabolic pathways (Wu et al. 2022; Zhao et al. 2018). At the community composition level, GLMP can promote the growth of beneficial bacteria, particularly *Bifidobacterium* and *Lactobacillus* genera. These are the bacteria that can utilize the carbohydrate moiety of GLMP, such as sialic acid, as a source of energy, thus having a competitive advantage in the gut. At the same time, GLMP may inhibit the colonization of pathogenic bacteria like *E. coli* and *Clostridium difficile* by not allowing them to adhere to the intestinal cell lining and by competing for nutrients. Combining action—enabling beneficial microbes and inhibiting pathogens—improves a more balanced and health‐inducing GM (Althnaibat et al. 2022; Koirala et al. 2024; Wu et al. 2021).

The composition and function of GM of very preterm newborns were evaluated with the effects of an infant formula that contained the particular prebiotic combination of the ratio of short‐chain GOS and *long‐chain fructo‐oligosaccharides* (9:1) and GLMP. The major species in the treatment group and the control group were *Klebsiella aerogenes* , *E. coli, Enterococcus fecium*, and *Klebsiella pneumoniae* . The treatment group's *Bifidobacterium* abundance dramatically increased following the 4‐week intervention. The findings indicated that the intestinal microecology of very preterm newborns benefits from formula enhanced with the ratio of short‐chain GOS and *long‐chain fructo‐oligosaccharides* (9:1) and GLMP (Lee et al. 2015; Yu et al. 2022). The effects of GLMP on satiety, glucose homeostasis, inflammation, and the fecal microbiota were assessed in 13 obese women in a dose‐finding crossover study. GLMP substantially increased the amylin concentration of the postprandial area under the curve compared to the soy meal tolerance test, and it was associated with higher satiety and C‐peptide. Consuming GLMP supplements three times a day decreased entire α diversity, while consuming them twice a day decreased members of the genus *Streptococcus* (Hansen et al. 2023).

The protective influence of GLMP on metabolic syndrome was analyzed; 36 male mice were separated into 3 groups of 12 mice, each with three different dietary conditions for 12 weeks. Mice were orally fed either a standard chow with water only or a high‐fat, high‐fructose diet with 5.2 kcal/g containing 15% protein, 20% sucrose, 65% fat, and 30% fructose in drinking water. One group of mice also orally received a high‐fat, high‐fructose diet with 200 mg/kg GLMP. The results indicated that GLMP‐treated animals showed a tendency toward a reduction in bile acids without any evident alterations in the composition of their intestinal flora. Given its positive effects on risk variables, including inflammation, oxidative stress, and endoplasmic reticulum stress, without affecting GM, GLMP provides significant promise for combating metabolic syndrome‐related components and effects (Sauvé et al. 2024). The impact of GLMP supplementation on GM of nine Phenylketonuria (PKU) patients was evaluated by comparing GM before and after the 6‐month intervention under 16S rRNA sequencing analysis. The particular prebiotic impact on the butyrate‐producing *Agathobacter* spp. and lesser influence on *Subdoligranulum* was found, but no significant alterations in GM were observed. The findings point to GLMP as a safe substitute in the PKU diet and its potential prebiotic function on some taxa without significantly altering the commensal GM (Montanari et al. 2022).

The systemic and intestinal immunomodulatory effects of casein GLMP taken orally were evaluated in 34 healthy Caucasians aged between 18 and 60 years, BMI of 18–25 kg/m^2^, and absence of lactose intolerance, milk protein allergy, and chronic disease. Comparing casein GLMP to a reference drink demonstrated no systemic or intestinal immunomodulatory effects on fecal calprotectin or the high‐sensitivity C‐reactive protein levels, fecal microbiota composition, or fecal SCFA content. There were no serious side effects from casein GLMP consumption, and it had no effect on body weight or satiety. There was no modification or induction of GI symptoms by casein GLMP (Wernlund et al. 2021).

## 4 Protective Mechanism of Whey Protein Against Gut Dysbiosis

### 4.1 Protein Fermentation

Once a particular WP fraction or whole protein content arrives in the colon, gut bacteria start to metabolize them via fermentation which results in the production of BCFAs and SCFAs. Propionic acid, butyric acid, and acetate are commonly produced SCFAs capable of promoting gut health (Aloo and Oh 2022; Sánchez‐Moya et al. 2017). Factors e.g., GM composition of individuals, consumed WPs content, and dietary intake influence the fermentation process and thus determine the resultant SCFA profiles. Metabolism of proteins also releases ammonia, sulfur‐containing metabolites like hydrogen sulfide and methanethiol, and other compounds like serotonin, phenethylamine, tryptamine, and histamine, which can exhibit negative impacts on gut health due to long‐term supplementation (Moreno‐Pérez et al. 2018).

Fermentation of WPs by lactic acid bacteria (LAB) reveals various peptides with different biological activities, nutritious properties and health benefits (Daliri et al. 2018; Mazorra‐Manzano et al. 2020). WP‐derived peptides exhibit growth‐promoting effects on *Bifidobacterium* and positively modulated GM, maintaining energy balance, metabolic activities and weight control. For instance, ALA hydrolysates can modulate GM and remarkably reduce weight gain (Li et al. 2019). Supplementation of WPH to infant diet significantly promoted the growth of LAB in infant gut and increased production of SCFAs (Feng et al. 2022). Besides, an enteral whey peptide diet showed a significant increase in the concentration of cecal SCFAs in comparison to standard diet (Tomoda et al. 2015). The pH is a critical factor in the production efficiency and variety of SCFAs during WPs digestion. The evidence suggests that WPs are beneficial for metabolism and positively modulate GM in infants and adults (Boscaini et al. 2023).

### 4.2 Antibiotic Effects

WPs have several fractions with well‐known antimicrobial properties, contributing to gut microbial balance. LF, LP, and GLMPs can inhibit the growth of harmful bacteria and pathogens while supporting probiotics in GM due to their antibacterial and prebiotic activities (Abdel‐Hamid et al. 2016; Gupta and Prakash 2017). LF can interfere with the metabolic process of the pathogens by depriving them of iron; LP can create reactive species with antimicrobial activities against pathogens; and Igs can suppress the colonization of pathogens in gut while supporting diverse beneficial GM (Gallo et al. 2024). Besides antioxidant and antihypertensive properties, whey BAPs have significant antibacterial activities capable of inhibiting various pathogens (Minj and Anand 2020). Complexation with metal ions can enhance bioavailability and antimicrobial efficacy of WPs in food formulations (Rodzik et al. 2020). WPH and peptide extracts have prebiotic activity and stimulate the growth of probiotic bacteria like *Lactobacillus* and *Bifidobacterium* as well (Liu and Chen 2023; Rackerby et al. 2024; Yu et al. 2016). A recent study revealed that WPs supplementation in diet significantly promoted the growth of beneficial bacteria in gut and regulating GM while showing anti‐photoaging activity after UV‐exposure (Wang, Zhou, et al. 2024). Besides, GLMPs and LF can increase probiotics such as *Bifidobacterium* and *Lactobacillus* due to their prebiotic action while suppressing *Enterobacteriaceae*, coliforms, *Eschericia* and *Shigella* in the intestines (Chen et al. 2012; Hu et al. 2019; Ma et al. 2023).

Antimicrobial properties of WPs and peptides are considered promising functional molecules in maintaining balance of GM, thus supporting gut health and immune system. For instance, BAPs of ALA have been expressed to promote GM modulation by acting as prebiotics to support the growth of probiotic species, besides exhibiting antimicrobial activity against various pathogens (Brumini et al. 2016; Kamau et al. 2010; Lajnaf et al. 2020; Layman et al. 2018).

### 4.3 Inflammatory Responses

Inflammatory responses in the body are associated with undesirable nutrition (processed foods, fats, etc.), lifestyle, and background health issues, which reveal irritations in the GI system and disturb the balance in the colon. Due to superior functionalities, proteins are suggested to be significant ingredients in diet to repair damaged intestinal tissue, enhance immunity and relieve inflammation, thus supporting colon health. Favorable effects of WPs against chronic colon inflammation have been demonstrated due to activation of some biochemical pathways inhibiting the generation of pro‐inflammatory markers, e.g., cytokines, chemokines and other mediators, and downregulation of some inflammation signaling pathways (Adler et al. 2023; Tunc et al. 2021). Although WPs have considerable health benefits including anti‐inflammatory effects, they are potentially associated with some inflammatory responses influencing GM in susceptible individuals. Major WPs like BLG and ALA are well‐known allergens for some individuals. Immunological responses to these WP fractions can trigger inflammation in digestive systems, revealing disturbed microbial balance in the gut (Cheng et al. 2023; Złotkowska et al. 2023). This situation may cause the proliferation of certain pathogens instead of beneficial gut microorganisms (Xu et al. 2025). Besides, the immune system of some sensitive individuals can exhibit aggressive responses if it perceives WPs as harmful molecules. This may arise potential dysbiosis and inflammation favoring pathogens and suppressing the beneficial diversity of GM.

The inflammation potentially induced by allergen proteins, especially in sensitive individuals, is also capable of extending intestinal permeability (Samadi et al. 2018), known as leaky gut referring to the displacement of bacterial toxins into the bloodstream, which causes a worsened inflammatory response in the gut and whole body (Ballegaard and Bøgh 2023; Poto et al. 2023). Increased toxin circulation can cause changes in the composition of GM, potentially resulting in favored pathogens and inflammatory bacteria (Yoo et al. 2020). Such inflammation stress can modify the fermentation process in the gut, revealing an altered metabolic path and thus diminished health‐promoting SCFA formation (Ney et al. 2023). Besides, new metabolites to be produced by inflammatory bacteria can further elevate inflammation, generating a closed loop maintaining dysbiosis.

Long‐term ingestion of BLG bound glycation products (common in thermally processed foods) has been found to trigger oxidative stress and inflammation significantly, leading to various outcomes including lipid metabolism perturbation, intestinal barrier dysfunction, negative changes in GM composition and a decrease in the SCFA content (Shi et al. 2023). Additionally, increased protein intake facilitates the reaching of undigested proteins to the colon, inducing fermentation by colonic GM and generation of some microbial‐derived metabolites like ammonia and phenolics, which then lower barrier functions of the intestines. On the basis of gender, this potentially reveals inflammation and impaired intestinal lining leading to leaky gut (James et al. 2024). Thus, excessively consumed WPs through imbalanced diets may also give rise to alterations in metabolic runs and negative modulation of GM. Since the ratio of *Firmicutes*/*Bacteroidetes* plays a crucial role in effecting intestinal homeostasis, the change in this ratio is strongly associated with dysbiosis, thus resulting in gut inflammation and respective disorders (Stojanov et al. 2020). Previous reports have also concluded on the detrimental effect of high protein diets on GM especially during extremely low carbohydrate intake for weight loss purposes (Farsijani et al. 2022). Hence, the negative effects of the high protein intake on GM could have arisen from different reasons; a careful evaluation can consider other factors including whole diet content, health records, medications, and daily routines of the individuals.

### 4.4 Consequences of Dysbiosis

**FIGURE 2:**
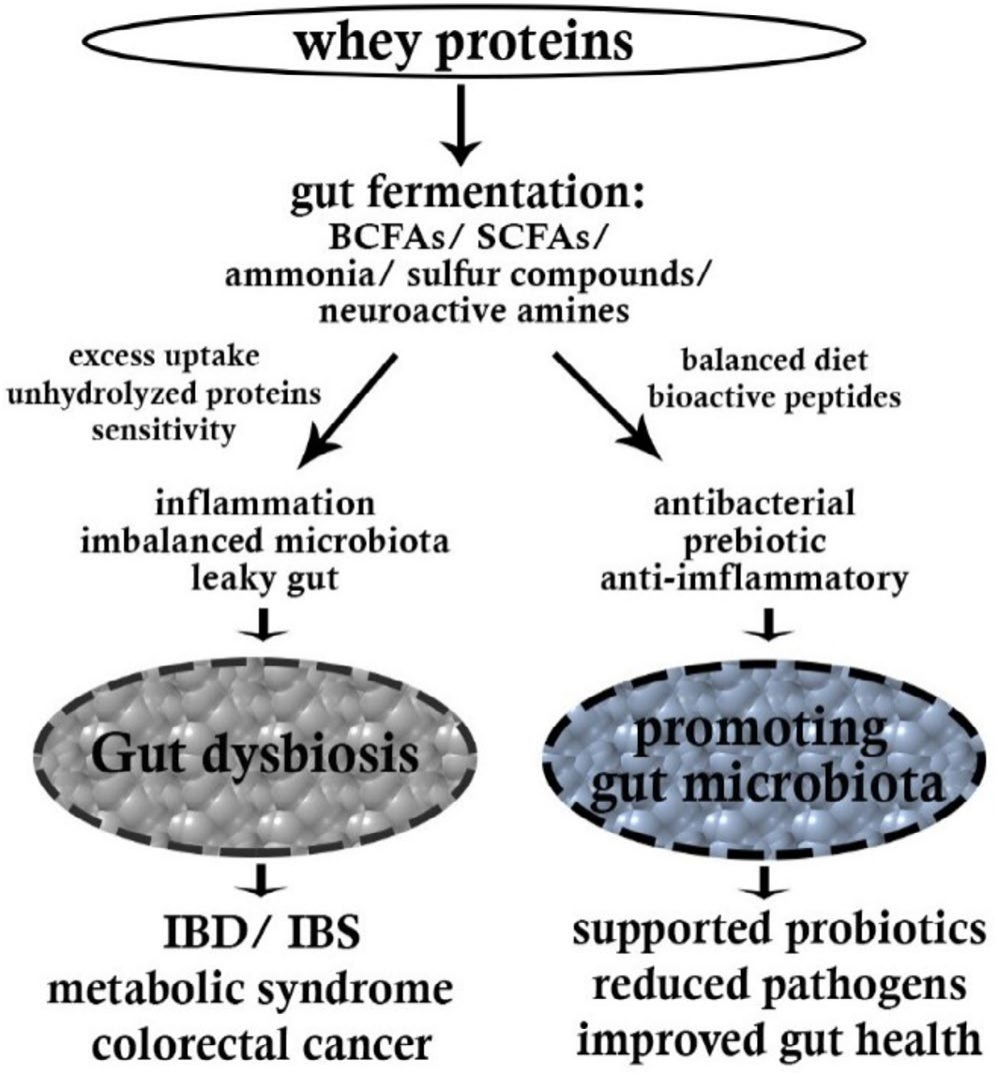
Mechanisms linking whey protein to gut dysbiosis.

The impact of WPs on gut health and dysbiosis is a complex fact depending on various factors, e.g., protein intake dose and period, GM composition and sensitivity of the individuals (Figure 2). Disruption and an imbalance in microbial content of the gut represent dysbiosis, which causes various health outcomes including metabolic disorders and severe diseases (Afzaal et al. 2022; Mostafavi Abdolmaleky and Zhou 2024). On the basis of the amount of dietary uptake, the bioactive content of WPs may alter the composition of GM, revealing altered diversity and abundance of some specific groups. Common findings indicate the increase in *Bifidobacteria* and *Lactobacilli* via WPs intake, while some studies declare that excess consumption can promote some pathogenesis as well, which potentially contribute to dysbiosis (Amaretti et al. 2019; Wu et al. 2022).

Allergenic fractions of WPs can influence the gut's immune response of allergenic individuals and result in inflammation. For some individuals, they can exhibit anti‐inflammatory effects, while for others, they can induce inflammation with shifting gut microbial populations. In the case of lactose intolerance and sensitivity to dairy proteins, WPs can aggravate irritation in the gut, leading to dysbiosis (Al‐Beltagi et al. 2022; Tagliamonte et al. 2023). Overexposure may intensify bloating, gas, diarrhea, and constipation in the bowels. Imbalanced GM can increase the risk of IBD and/or irritable bowel syndrome (IBS), consequently. Especially, damages occurring in the intestinal barrier due to dysbiosis led to an increase in intestinal permeability, allowing the passage of some gut content through the blood system, which then stimulates systematic inflammation and overreaction of the immune system, resulting in IBD, IBS, and metabolic disorders (DeGruttola et al. 2016). Even the inflammation itself can damage the colon tissues and provoke these syndromes. Additionally, imbalanced GM can be associated with a lack of SCFA production, thus possibly promoting pro‐inflammation in the bowel and conditioning to the mentioned disorders (Machiels et al. 2014; Venegas et al. 2019).

Lond term diets with high protein content like WPs can also influence metabolic pathways in the body. They may impair lipid and glucose metabolism, which give rise to metabolic diseases including insulin resistance, obesity, and diabetes (Cai et al. 2022). Besides, the shift in GM due to high intake of proteins may impair the gut's function in promoting energy balance (Madsen et al. 2017). Some evidence has indicated that diets with high content of WPs can lower the production of SCFAs when the proteins are replaced with fiber‐rich ingredients (Xu, Curtasu, et al. 2020). This may contribute to an imbalanced GM in the colon, causing metabolic dysfunctions and life‐threatening diseases like colorectal cancer, the most common form of cancer worldwide (Yang and Yu 2018). Dysbiosis is thought to promote chronic inflammation in the colon and rectum, modifying the metabolic paths and generating carcinogenic metabolites (Sun and Kato 2016). Dysbiosis‐related alterations in gut permeability may lead to activated immune responses and extended inflammation, as well as the development of some bacterial species like *Fusobacterium* capable of promoting metastasis in colorectal cancer (Kostic et al. 2012; Wu et al. 2022).

## 5 Colonic Health Implications

### 5.1 Inflammation and Gut Health

GMd plays a pivotal role in the development and progression of colonic inflammation, particularly in IBD such as Crohn's disease and ulcerative colitis. Dysbiosis is marked by a reduction in beneficial microbes and an overgrowth of pro‐inflammatory taxa. For instance, *Faecalibacterium prausnitzii* , a known butyrate producer with anti‐inflammatory properties, is significantly reduced in Crohn's disease patients. Sokol et al. (2008) found that lower levels of *F. prausnitzii* were associated with elevated levels of IL‐6 and TNF‐α, key inflammatory cytokines. Gevers et al. (2014) further demonstrated that treatment‐naïve pediatric Crohn's disease patients exhibited a distinct microbial signature characterized by reduced diversity and increased abundance of *Enterobacteriaceae*, suggesting that microbial imbalance may precede clinical symptoms. This is supported by animal studies such as Sellon et al. (1998), where IL‐10 knockout mice developed spontaneous colitis only in the presence of GM, indicating a causal role for dysbiosis in initiating inflammation.

These microbial shifts compromise the intestinal barrier, allowing microbial products like LPS to activate toll‐like receptors (TLRs), particularly TLR4, on immune cells. This activation triggers a cascade of pro‐inflammatory responses, perpetuating mucosal damage and chronic inflammation (Sartor 2008). These findings underscore the importance of targeting GM composition to mitigate inflammation in IBD.

### 5.2 Microbial Diversity and Disease Prevention

Microbial diversity is essential for maintaining gut homeostasis and preventing disease. A diverse GM contributes to metabolic flexibility, competitive exclusion of pathogens, and the production of SCFAs, especially butyrate. Butyrate supports colonocyte health, reinforces tight junctions, and modulates immune responses through G‐protein‐coupled receptors and histone deacetylase inhibition (Canani et al. 2011). Manichanh et al. (2006) reported that IBD patients had significantly reduced microbial richness and evenness compared to healthy individuals. Similarly, Qin et al. (2010) found that individuals with type 2 diabetes had a less diverse GM with impaired butyrate synthesis pathways, linking microbial diversity to metabolic and inflammatory diseases. Therapeutic strategies aimed at restoring microbial diversity have shown promise. Paramsothy et al. (2017) conducted a randomized controlled trial using multi‐donor fecal microbiota transplantation (FMT) in ulcerative colitis patients. The intervention led to clinical remission, increased microbial diversity, and elevated SCFA levels, highlighting the therapeutic potential of GM modulation. Low microbial diversity in the gut has been increasingly associated not only with digestive and immune‐related issues but also with a broader range of health concerns. Studies have linked reduced gut microbial diversity to obesity, allergies, and even neurological conditions such as anxiety and depression. Including this context highlights the far‐reaching implications of gut health and may help underscore the importance of microbial diversity in maintaining overall well‐being.

### 5.3 Potential Therapeutic Approaches: Role of Whey Proteins

WPs have been shown to beneficially modulate GM composition and function, offering a promising dietary approach to support colonic health. Table 2 presents a summary of key studies on WPs and colonic health. In a DSS‐induced colitis model, Sprong et al. (2010) demonstrated that rats fed with WPI had higher levels of *Lactobacillus* and *Bifidobacterium*, along with reduced colonic inflammation and lower expression of pro‐inflammatory cytokines. This suggests that WPs can enhance beneficial microbial populations and suppress inflammatory responses. Zimecki and Artym (2005) reported that LF binds LPS, preventing TLR4 activation and downstream inflammatory signaling, thereby protecting the colonic mucosa. Human studies also support these findings. Kriss et al. (2019) found that WPs supplementation in older adults increased microbial diversity and SCFA concentrations, particularly butyrate, which correlated with improved gut barrier function. Requena et al. (2010) showed that fermented whey products enhanced *Bifidobacterium* abundance and reduced fecal calprotectin, a marker of intestinal inflammation. Additionally, cysteine‐rich WPs support glutathione synthesis, a key antioxidant that protects colonic epithelial cells from oxidative stress. Bounous and Gold (1991) highlighted the role of WPs in enhancing cellular antioxidant defenses, which is particularly relevant in IBD where oxidative damage exacerbates mucosal injury. Collectively, these studies suggest that WPs not only reduce colonic inflammation but also promote microbial diversity—an essential factor in maintaining gut health and preventing relapse in dysbiosis‐associated conditions.

**TABLE 2:** Key studies on whey proteins and colonic health.

Model	Intervention	Key findings	References
Human	N/A	Lower *F. prausnitzii* linked to higher IL‐6 and TNF‐α in Crohn's disease	Sokol et al. (2008)
		Reduced diversity and increased pro‐inflammatory taxa in pediatric Crohn's disease	Gevers et al. (2014)
	Whey protein supplementation	Increased diversity, SCFAs, and improved gut barrier markers in older adults	Kriss et al. (2019)
	N/A	IBD patients showed reduced microbial richness and evenness	Manichanh et al. (2006)
		Type 2 diabetes linked to less diverse microbiota and reduced butyrate production	Qin et al. (2010)
	FMT	FMT improved remission in UC, increased diversity and SCFA levels	Paramsothy et al. (2017)
	Fermented whey products	Enhanced *Bifidobacterium*, reduced fecal calprotectin (inflammation marker)	Requena et al. (2010)
Animal	N/A	Dysbiosis precedes inflammation in IL‐10 knockout mice.	Sellon et al. (1998)
	Whey protein isolate	Increased *Lactobacillus*, *Bifidobacterium*; reduced inflammation in colitis model	Sprong et al. (2010)
In vitro	Lactoferrin	Antimicrobial, promotes beneficial microbes, binds LPS to reduce inflammation	Zimecki and Artym (2005)
	Cysteine‐rich whey proteins	Boosted glutathione synthesis, protected colonocytes from oxidative stress	Bounous and Gold (1991)

## 6 Conclusions

This review highlights the intricate interplay between WPs, GM balance, and colonic health. WPs, rich in BAPs, possess antimicrobial, antioxidant, and immunomodulatory properties that can positively influence GM composition and barrier integrity. These attributes suggest a therapeutic potential for WPs in mitigating GMd—a condition linked to colonic inflammation, immune dysregulation, and increased risk of colorectal pathologies. Emerging evidence supports WP‐derived compounds, such as GLMPs and LF, as modulators of microbial ecology with prebiotic‐like effects. These shifts correlate with enhanced SCFA production, tightened intestinal barrier integrity, and reduced inflammation, offering protection against colitis, colorectal cancer, and metabolic disorders. Mechanistically, WPs counteract dysbiosis‐driven pathologies by inhibiting LPS translocation, downregulating pro‐inflammatory cytokines (e.g., TNF‐α), and promoting mucin synthesis. However, despite promising in vitro and animal data, the translational relevance to human colonic health remains underexplored.

Critical research gaps persist. Most existing studies are preclinical, short‐term, limited in scope, and rarely account for individual GM variability. There is a lack of robust, controlled clinical trials investigating the long‐term impacts of dietary WPs on human GM composition and colonic outcomes, especially in populations with IBD or colorectal cancer risk. The role of WP‐derived phages in microbial regulation remains underexplored, as do synergistic interactions with fibers or probiotics. Additionally, population‐specific GM diversity necessitates personalized approaches to maximize therapeutic benefits. Future research should prioritize large‐scale, longitudinal clinical trials and placebo‐controlled studies with microbiome sequencing and inflammatory markers to validate WP's prophylactic and therapeutic efficacy. Additionally, mechanistic studies are needed to delineate the specific bioactive components of WP that confer gut‐modulatory benefits. It should also dissect strain‐specific effects of whey peptides and their epigenetic impacts on host‐microbe crosstalk. Integrating multi‐omics (metagenomics, metabolomics) will elucidate context‐dependent outcomes, while standardized protocols for WP formulations are essential for reproducibility. Addressing these gaps will be crucial to harnessing WPs as functional dietary tools for colonic health optimization and targeted interventions in precision nutrition.

## Author Contributions

Conceptualization: T.J.A. Writing: T.J.A., C.‐C.L., O.T. and A.R. Review, editing, and final draft: T.J.A. and S.M.J. Verification: S.M.J.

## Ethics Statement

The authors have nothing to report.

## Conflicts of Interest

The authors declare no conflicts of interest.

## Data Availability

No new data were generated in this manuscript.
